# Cold Storage and Drying Alter Polar Metabolite Profiles in Commercial Sprouts of Eight Plant Species

**DOI:** 10.3390/molecules31142442

**Published:** 2026-07-12

**Authors:** Lesław Bernard Lahuta, Karolina Stałanowska, Marcin Horbowicz

**Affiliations:** Department of Plant Physiology, Genetics and Biotechnology, University of Warmia and Mazury in Olsztyn, Oczapowskiego Street, 1A/103, 10-719 Olsztyn, Poland; karolina.stalanowska@uwm.edu.pl

**Keywords:** sprout, cold storage, drying, polar metabolites, sucrose, proline, raffinose, GABA

## Abstract

The application of the gas chromatography coupled to mass spectrometry (GC-MS) has been applied for metabolite profiling in sprouts of *Brassicaceae* (broccoli, kale, radish), sunflower, and *Fabaceae* (alfalfa, clover, lentil, and mung bean). Soluble carbohydrates were quantitatively the major fraction of total polar metabolites in *Brassicaceae* and sunflower sprouts. The main sugars in sunflower, mung bean, and *Brassicaceae* sprouts were fructose and glucose, while in clover, alfalfa, and lentil sprouts, the dominant sugar was sucrose. *Myo*-inositol was found in the sprouts of all analyzed species, while d-*chiro*-inositol, *epi*-inositol, or *scyllo*-inositol, as well as d-pinitol, were present in the sprouts of some legumes. Among the amino acids in clover and alfalfa sprouts, asparagine, serine, and threonine were quantitatively dominant. During cold storage of sprouts or their drying, a decrease in the content of monosaccharides and an increase in the levels of sucrose were found. Furthermore, such conditions of sprouts storage caused an accumulation of raffinose, proline, and *γ*-aminobutyric acid. The reduction in the content of monosaccharides and the increase in the content of free amino acids in dried sprouts is new information. Moreover, information about the small effects of low-temperature storage and drying of sprouts on the content of cyclitols seems to be valuable.

## 1. Introduction

The process of seed germination is an important method for increasing the nutritional value of food, as the resulting sprouts have more beneficial dietary properties than seeds [1]. Sprouting is a simple, quick, and inexpensive process that does not require sophisticated equipment and produces relatively high yields [2]. In recent years, consumer interest in edible plant sprouts has been growing due to their nutritional and health-promoting properties [3,4,5]. During the germination process, storage compounds present in seeds, such as protein, starch, and fat, are decomposed, resulting in a rapid increase in the content of free amino acids, low molecular weight sugars, glycerol, and fatty acids, as well as increasing their bioavailability and nutritional utilization [1,4,6]. Furthermore, the hydrolysis of antinutritional compounds, including lectins, protease inhibitors, raffinose oligosaccharides, and phytin, increases the nutritional value of sprouts compared to dry seeds [6]. At the same time, new secondary metabolites are formed, leading to an increase in antioxidant activity of the sprouts [7] and their anti-inflammatory, anti-diabetic, anti-cancer, and neuroprotective properties [1,3,8,9,10]. Thus, sprouts are suitable for promoting their use in functional foods and dietary interventions [11]. So far, the most popular plant species whose germinated seeds and sprouts are used as food for direct consumption are species of the *Brassicaceae* genus [12,13] and *Fabaceae* [6,10,14].

*Brassicaceae* sprouts are an important type of food due to their rich composition of bioactive compounds compared to adult plant tissues. Depending on the conditions and time of germination, the sprouts of these species may contain two to 10 times more phytochemicals than the original seeds [15]. Typically, seven- or eight-day-old *Brassicaceae* sprouts are ready for harvesting, processing, and commercialization, maintaining phytochemical content at a higher level than in other vegetables [12,16,17]. Important and characteristic compounds found in sprouts of these plants are glucosinolates [18]. *Brassicaceae* sprouts also contain numerous phenolic compounds, such as phenolic acids and flavonoids, and exhibit high antioxidant capacity [19,20,21]. *Brassicaceae* sprouts, when crushed, have a spicy and pungent taste resulting from the enzymatic breakdown of glucosinolates into isothiocyanates during consumption [22].

*Fabaceae* seeds are an inexpensive source of protein and can be a meat substitute for vegetarians [23]. However, seeds of this family are generally difficult to digest. The sprouting process removes many nutritionally unfavorable components, while macromolecules undergo enzymatic hydrolysis into simpler forms or new compounds [24]. Antinutritional substances present in legumes include tannins, saponins, oligosaccharides, phytic acid, and protease inhibitors, which reduce the bioavailability of proteins, vitamins, and minerals [6,25,26]. The most popular species used to produce sprouts are soybean, mung bean, lentils, and alfalfa.

Sunflowers (*Helianthus annuus* L.) are cultivated worldwide for their nutritional and medicinal value. Sunflower seeds are both a source of valuable edible oil and a popular snack in Europe and Asia [27]. Sunflower sprouts, like those of other species, have higher antioxidant activity than its seeds, primarily due to the increased content of phenols, melatonin, and isoflavones following germination [28].

Until consumption, sprouts are stored in refrigerated conditions, both in supermarkets and at home. Data on the effect of cold storage and drying processes on compounds contained in sprouts are limited and mainly concern secondary metabolites. It is known that prolonged low temperatures can affect their nutritional value, such as levels of vitamins and other sensitive nutrients [29]. Furthermore, long-term storage can lead to water loss and slow drying of the sprouts. For instance, the results obtained during the storage of radish sprouts showed that the storage temperature significantly influenced the product quality and shelf life [30]. The composition and nutritional quality of legumes are affected by sprouting and further storage at low temperatures [26]. In soybean sprouts, the isoflavone content after 7 days of storage at 4 °C was generally equal to or higher than in the sprouts before storage. In turn, storage of cabbage sprouts in the refrigerator for more than 7 days resulted in a significant decrease in the level of glucosinolates, especially glucoraphanin and sinigrin [31].

Drying is one of the most important methods of food preservation. The quality of dried sprouts is determined on the basis of the drying conditions and methods used, which must be selected in such a way as to preserve bioactive compounds and their nutritional value [32]. Both heat-drying and freeze-drying were shown to reduce phytochemical content compared to fresh alfalfa and flax sprouts [32]. However, freeze-drying allowed better preservation of phytoestrogens, phytosterols, and total tocols compared to heat-drying [32,33].

Since plant sprouts contain high levels of secondary metabolites, most of the previously published papers on sprouts concerned the content of these compounds and their changes under the influence of various factors [34,35]. Many of these reports concern the effect of different elicitors on the phytochemical composition of sprouts [20,36,37,38,39,40]. However, changes in the primary metabolite profile have a significant impact on secondary metabolism. Metabolite profiling analysis based on liquid chromatography (LC) or gas chromatography (GC), combined with advanced identification methods using mass spectrometry (MS), seems to be a suitable method for comprehensive studies of plant-based foods [41,42,43,44,45,46,47,48,49].

In our previous study, we demonstrated the usefulness of GC-MS for analyzing the changes in the metabolic profile of polar compounds during germination of pea, cucumber, and wheat [46]. The aim of the study herein was to compare the profiles of primary metabolites in sprouts of eight commercially available plant species and the changes that occur in them as a result of storage at cold temperature and drying in mild conditions.

## 2. Results

### 2.1. Identification of Polar Metabolites in Sprouts of Eight Species

A total of 50 polar metabolites were identified in the analyzed sprouts (Table S1). Comparing the GC chromatograms of the sprouts of the assessed plant species, it can be seen that the profiles of polar metabolites of *Brassicaceae* species are similar (Figure S1). A similar assessment can be demonstrated among the sprouts of the *Fabaceae* family (Figure S2) and sunflower (Figure S1). *Brassicaceae* sprouts contained 42 polar compounds, including 11 organic acids, 16 proteinogenic amino acids, three non-proteinogenic amino acids, 10 sugars and sugar acids, glycerol, and phosphoric acid (Table 1). Sunflower sprouts were found to contain 31 metabolites (seven organic acids, 14 proteinogenic amino acids, two non-proteinogenic amino acids, six sugars, glycerol and phosphoric acid), whereas 41 were in *Fabaceae* (seven organic acids; 15 proteinogenic amino acids; three non-proteinogenic amino acids; 14 sugars, sugar acids, and cyclitols; glycerol; and phosphoric acid) (Table 1).

In addition to primary metabolites common for all species, i.e., malic acid, citric acid, glucose, fructose, sucrose, *myo*-inositol, and most of the found proteinogenic amino acids, including γ-aminobutyric acid (GABA), some metabolites were species-specific. Sprouts of *Fabaceae* contained homoserine (Table 1), while *myo*-inositol isomers, *epi*- and *scyllo*-inositol, were found in mung bean, or d-*chiro*-inositol and its methyl derivative, d-pinitol, were in alfalfa, clover, and lentil (Table 2). Moreover, in species containing d-pinitol, its di-galactoside (ciceritol) was also found (Table 1). Additionally, in sprouts of broccoli, kale, sunflower, and mung bean, hydroxyproline was identified (Table 1).

### 2.2. Polar Metabolite Profiles in Fresh Sprouts

**Figure 1:**
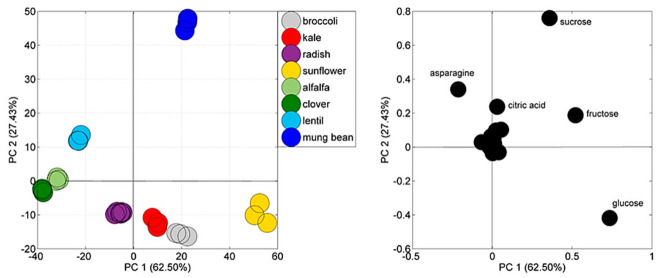
PCA score (**left**) and loading plots (**right**) of polar metabolic profiles of examined sprouts of eight plant species.

In control sprouts, the concentrations of polar metabolites differed among the investigated species. According to principal component analysis (PCA), samples of broccoli, kale, and sunflower were located in the bottom-right corner of the PCA plot, while samples of radish were close to kale but on the left side of PC1 (62.50% of variance, Figure 1).

The samples of alfalfa, clover, and lentil were on the left side of PC1, whereas the mung bean was separated and located in the top-right corner of the plot. Discrimination of samples was mainly dependent on differences in the concentrations of major primary metabolites: sugars (glucose, fructose, and sucrose), asparagine, and citrate. The first two principal components (PC1 and PC2) explained 62.50% and 27.43% of the total variance, respectively, accounting for 89.93% of the cumulative variance.

Conducting PCA based only on sugars confirmed the above differentiation of samples. Moreover, in *Fabaceae*, an additional metabolite separating the samples turned out to be d-pinitol, which was absent in mung bean (Figure S3). Additionally, PCA based only on proteinogenic amino acids clearly separates samples of *Fabaceae* from those of *Brassicaceae* due to the differences in asparagine and lysine (Figure S3). In control sprouts, the concentration of total polar metabolites (TPMs) was the highest in mung bean and sunflower (219.8 and 185.7 mg/g DW, respectively) and lowest in clover and radish (64.55 and 73.45 mg/g DW, Table S2).

In cruciferous and sunflower sprouts, the largest quantitative part was soluble carbohydrates, constituting 72.37–82.69% of TPMs, and in mung beans and lentils, approximately 58% of TPMs (Table S1). In the sprouts of *Brassicaceae* species, sunflower, and mung bean, the main sugars were glucose and fructose, and in clover, alfalfa, and lentil, it was sucrose (Table 1).

Sprouts of alfalfa and clover contained 2- and 4-fold more amino acids than soluble carbohydrates (Table S2). Among the amino acids, asparagine dominated in lentil and mung bean (Table 1). In *Brassicaceae*, the major amino acids were proline, lysine, and threonine, while these were glutamate, proline, and asparagine in sunflower (Table 1). In all species, besides GABA and β-alanine, other non-proteinogenic amino acids were also found—hydroxyproline in *Brassicaceae* or homoserine in *Fabaceae* (except mung bean).

In broccoli, kale, and sunflower sprouts, the main organic acids were malic acid, while in legumes and radishes, citric acid was the main organic acid. Additionally, the sprouts contained phosphoric acid and glycerol (Table 1).

### 2.3. Effect of Cold Storage Sprouts or Its Drying on Metabolite Profiles

Cold storage did not show any visible tissue damage of sprouts of all species. However, the drying process drastically affected their appearance due to twisting of the roots and hypocotyls, deformation of the cotyledons, and a change in the color of the roots and cotyledons to brown (Plate S1). After cold storage and drying, the concentrations of most metabolites in sprouts of particular species changed significantly (Tables S3–S5). Cold storage and drying increased the TPM content in broccoli and radish sprouts due to the accumulation of soluble carbohydrates. The opposite effect of both factors was observed in mung bean sprouts, in which TPMs decreased both after cold storage and drying (Table S2). Moreover, in sunflower and lentil, TPMs increased only after cold storage, while for kale, alfalfa, and clover, this was in response to drying. In both cases, this was mainly due to the accumulation of carbohydrates and amino acids. Cold storage increased TOA concentrations in broccoli and kale sprouts, while it decreased TOAs in alfalfa, clover, and mung bean sprouts. A reduction in TOA content was also demonstrated after drying alfalfa, lentil, and mung bean sprouts. (Table S2).

**Figure 2:**
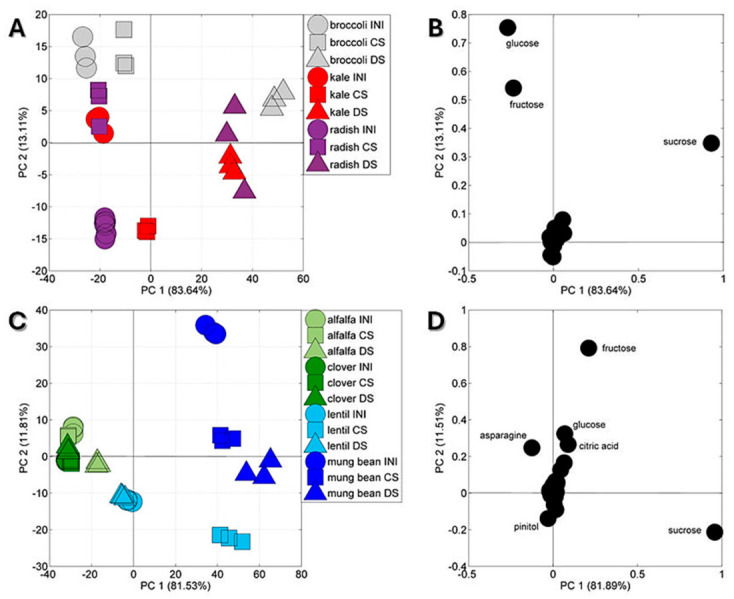
PCA scores (**A**,**C**) and loading plots (**B**,**D**) of the polar metabolic profiles of *Brassicaceae* (broccoli, kale, and radish; (**A**)) and *Fabaceae* (alfalfa, clover, lentil, and mung bean; (**B**)) sprouts. Symbols: circles, control (initial, INI); squares, after cold storage (CS); and triangles, after drying (DS).

Both cold storage and drying had various effects on the metabolic profiles in the sprouts of the examined species (Figure 2).

Samples of dried *Brassicaceae* sprouts were located on the right side of PC1 (83.64% of variance), while control and cold storage were on the left (Figure 2A). The first two principal components (PC1 and PC2) explained 83.64% and 13.11% of the total variance, respectively, accounting for 96.75% of the cumulative variance. It is worth noting that the PCA clearly differentiated the control samples of sprouts of individual species from those after cold storage and drying. (Figure S4). Control sprouts and sprouts after cold storage were located on the left side of PC1 (sharing 98.21 %, 88.53 %, and 87.11 % of variance for broccoli, kale, and radish, respectively), in the top or bottom corners. In contrast, samples of dried sprouts were located on the right side (Figure S4A,C,E). Similar sample discriminations were found in sunflower (Figure S4G). The main differentiating metabolites in these four species were sucrose, glucose, and fructose, as well as succinic acid in broccoli and kale (Figure S4B,D) or glutamic acid in radish and sunflower (Figure S4F,H).

The results of the analyses of alfalfa and clover sprouts, both the control samples and the samples after cold storage or drying, were close to each other on the left side of PC1 (81.53% of variance), while the results of the analyses of mung bean sprouts were on the right side (Figure 2C). In turn, the control and dried lentil sprout samples were located below PC2 (11.81%) and on the left, while the cold-stored samples were on the right side of PC1. (Figure 2C). The PC1 and PC2 explained 81.53% and 11.81% of the total variance, respectively, accounting for 93.34% of the cumulative variance. PCA for sprouts of each *Fabaceae* species separately showed clear discrimination in all samples (Figure S5A,C,E,G). The major metabolites discriminating the samples of *Fabaceae* species were asparagine and sucrose (Figure S5B,D,F,H).

#### 2.3.1. Soluble Carbohydrates

**Figure 3:**
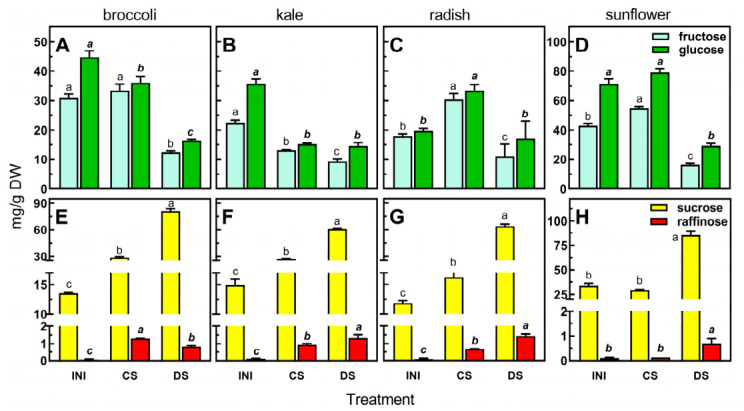
The concentration of fructose, glucose (**A**–**D**), sucrose, and raffinose (**E**–**H**) in sprouts of *Brassicaceae* (broccoli, kali, and radish; (**A**–**C**) and (**E**–**G**) and sunflower (**D**,**H**) before (initial, INI) and after cold storage (CS) or after drying (DS). The same letters above the bars indicate no significant differences (*p* < 0.05) after ANOVA and Tukey’s test. Abbreviation: DW—dry weight.

**Figure 4:**
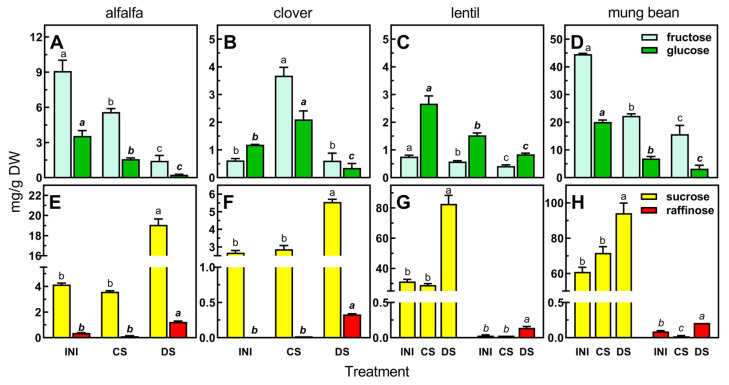
The concentration of fructose, glucose (**A**–**D**), sucrose, and raffinose (**E**–**H**) in sprouts of *Fabaceae* (alfalfa, clover, lentil, and mung bean) before (initial, INI) and after cold storage (CS) or after drying (DS). The same letters (a–c above the bars indicate no significant differences (*p* < 0.05) after ANOVA and Tukey’s test. Abbreviation: DW—dry weight.

In sprouts of *Brassicaceae* species and sunflower, the levels of glucose and fructose were higher than in legume sprouts but drastically decreased during their drying (Figure 3 and Figure 4).

Simultaneously, tissues accumulated sucrose (up to 60–85 mg/g DW) and noticeable amounts of raffinose. Sucrose and raffinose were also accumulated during the drying of *Fabaceae* sprouts (Figure 4). However, the levels of sucrose in alfalfa and clover were much lower than that in *Brassicaceae*, lentil, and mung bean (Figure 3 and Figure 4). Additionally, the drying process enhanced the concentration of *myo*-inositol in *Brassicaceae* and sunflower (Tables S3 and S4).

Cold storage also resulted in the accumulation of sucrose and raffinose in *Brassicaceae* sprouts (Figure 3), and sucrose in mung beans (Figure 4H). However, unlike the drying process, a reduction in monosaccharide content was only demonstrated in kale and mung bean sprouts (Figure 3B and Figure 4D). Cold storage of radish, sunflower, and clover sprouts increased the glucose and fructose content, but decreased these monosaccharides in alfalfa and lentil (Figure 4).

Sprouts of all examined species contained *myo*-inositol, while d-pinitol was found in measurable amounts only in alfalfa, clover, and lentil sprouts (Table 2). d-Pinitol was found in particularly high concentrations in lentil sprouts. In turn, d-*chiro*-inositol was found in alfalfa and lentil sprouts. In addition, *epi*-inositol, rarely found in plant tissues, was found in lentil and clover sprouts, while *scyllo*-inositol was found in mung bean and traces in clover sprouts. *Brassicaceae* sprouts did not contain measurable contents of d-pinitol, d-*chiro*-inositol, or *epi*- and *scyllo*-inositols (Table 2).

The highest cyclitol contents were found in lentil sprouts, containing high levels of d-pinitol, as well as *myo*-inositol, *epi*-inositol, and d-*chiro*-inositol (Table 2). Both cold storage and drying resulted in an increase in *myo*-inositol content in *Brassicaceae* and sunflower sprouts (Table 2). However, the drying process decreased the *myo*-inositol content in sprouts of alfalfa, clover, and mung bean. Cold storage, on the other hand, decreased *myo*-inositol content in mung beans and alfalfa, and reduced the level of d-pinitol in lentil. In turn, the sprout drying process had no effect on the content of this cyclitol in lentil, alfalfa, and clover (Table 2). In addition, both cold storage and drying increased the content of d-*chiro*-inositol in alfalfa sprouts, while cold storage also decreased the *scyllo*-inositol content in mung bean (Table 2). Moreover, the *epi*-inositol level did not change after cold storage or drying of lentil sprout.

#### 2.3.2. Amino Acids

**Figure 5:**
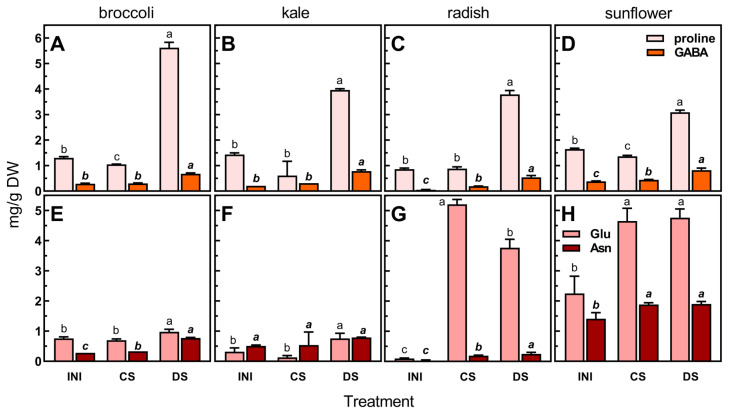
The concentration of proline, GABA (**A**–**D**), glutamic acid (Glu), and asparagine (Asn, **E**–**H**) in sprouts of *Brassicaceae* (broccoli, kale, and radish; (**A**–**C**) and (**E**–**G**)) and sunflower (**D**,**H**) before (initial, INI) and after cold storage (CS) or after drying (DS). The same letters (a–c above the bars indicate no significant differences (*p* < 0.05) after ANOVA and Tukey’s test. Abbreviation: DW—dry weight.

**Figure 6:**
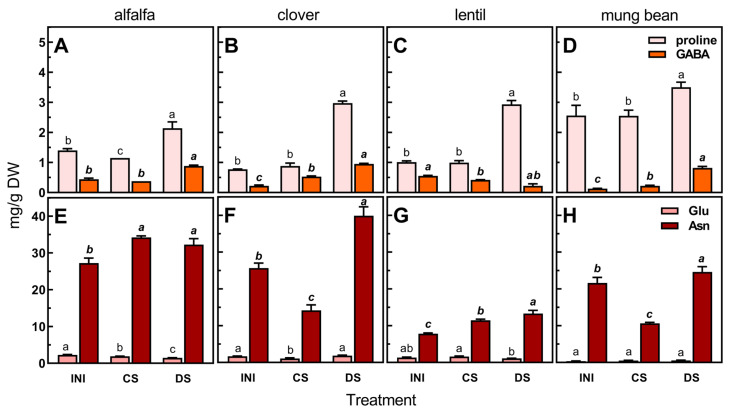
The concentration of proline, GABA (**A**–**D**), glutamic acid (Glu), and asparagine (Asn, (**E**–**H**)) in sprouts of *Fabaceae* (alfalfa, clover, lentil, mung bean) before (initial, INI) and after cold storage (CS) or after drying (DS). The same letters (a–c above the bars indicate no significant differences (*p* < 0.05) after ANOVA and Tukey’s test. Abbreviation: DW—dry weight.

Both drying and cold storage of sprouts affected the concentrations of most amino acids. However, only a few of them underwent similar changes in the sprouts of all eight species. In response to drying, sprouts of all species accumulated proline, GABA, and asparagine, but this was to a lesser extent in sprouts of the *Brassicaceae* family than in those of sunflower and *Fabaceae*. In turn, glutamic acid increased after drying in radish, sunflower, and *Fabaceae* sprouts (Figure 5 and Figure 6).

Cold storage had no significant effect on the concentration of proline in sprouts all species, while in radish and sunflower, it enhanced the content of GABA and glutamic acid (Figure 5G,H). In *Fabaceae*, a significant increase in asparagine was noted in alfalfa and lentil sprouts (Figure 6E,G).

#### 2.3.3. Organic Acids and Other Compounds

**Figure 7:**
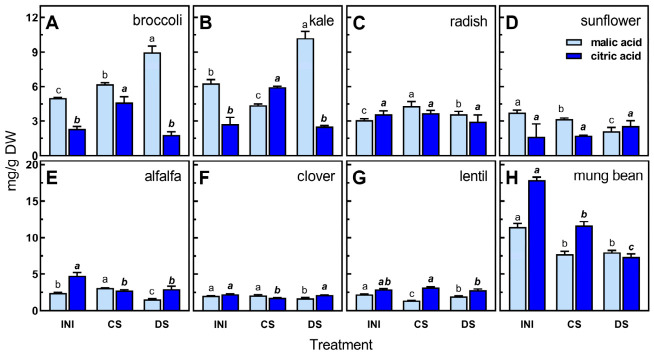
The concentration of malic acid and citric acid in sprouts of *Brassicaceae* (**A**–**C**), sunflower (**D**), and *Fabaceae* (**E**–**H**) before (initial, INI) and after cold storage (CS) or after drying (DS). The same letters (a–c) above the bars indicate no significant differences (*p* < 0.05) after ANOVA and Tukey’s test. Abbreviation: DW—dry weight.

The main organic acids in the examined sprout species were malic and citric acids, while phosphoric acid and glycerol were also detected among other compounds. In broccoli and kale sprouts, the content of malic and citric acids increased after both drying and cold storage (Figure 7A,B). Cold storage also increased the level of malic acid in radish and alfalfa sprouts (Figure 7A,C,E). However, in mung bean sprouts, both drying and cold storage reduced the levels of malic and citric acids in sunflower and lentil (Figure 7D,G,H).

The concentration of phosphoric acid significantly increased after drying sprouts of *Brassicaceae* plants (Table S3), sunflower (Table S4), clover, and mung bean (Table S5). However, the process decreased the glycerol content in sprouts of *Brassicaceae* plants, sunflower, alfalfa, and mung bean.

Drying or cold storage of sprouts had only a minor effect on the concentration of specific polar metabolites, such as hydroxyproline, which was increased by both factors in broccoli and kale sprouts (Table S3) and in mung beans after drying only (Table S5). In contrast, the content of homoserine was not changed in *Fabaceae* sprouts, except for a decrease in its content in clover sprouts after cold storage (Table S5).

## 3. Discussion

Most of the numerus studies on the phytochemical composition of sprouts concern secondary metabolites, especially phenolic acids and flavonoids [20,28,33,34,35,36,37,38,39,40,47]. In the case of *Brassicaceae* sprouts, in addition to phenolic compounds, species-specific compounds such as glucosinolates and isothiocyanates were also studied [16,19,20,21,48,49]. The results of analyses of primary compounds in sprouts and young seedlings are few [8,50,51,52,53]. This paper presents the results of GC/MS analyses of carbohydrates, amino acids, organic acids, and other polar compounds in the sprouts of eight plant species most commonly available on the market. Analyses were carried out on sprouts of four *Fabaceae* species (alfalfa, clover, lentil, mung bean), three *Brassicaceae* species (radish, broccoli, kale), and sunflower. A similar method was used to evaluate metabolic changes during germination of mung bean, lentil, and four *Lupinus* species [48,52].

### 3.1. Metabolic Profiles of Fresh Sprouts

*Brassicaceae* and sunflower sprouts contained higher content of monosaccharides than *Fabaceae* sprouts, except mung bean (Figure 3 and Figure 4). Among these, glucose was the major monosaccharide in broccoli, kale, sunflower, and lentil sprouts, while this was fructose in alfalfa and mung bean sprouts. These results confirm the data recently published by Balik et al. [53], who showed higher glucose content than fructose in broccoli, radish, and lentil sprouts. Previously, Wojdyło et al. [8] also showed that glucose contents were higher than fructose in radish and broccoli sprouts but not in sunflower and lentil sprouts. In mung bean, lentil, and clover sprouts, the main soluble carbohydrate was sucrose (Figure 3 and Figure 4).

Sprouts of all tested species contained *myo*-inositol, whereas d-pinitol was found in measurable amounts only in alfalfa, clover, and lentil sprouts (Table 2). In addition, d-*chiro*-inositol (DCI) was found in small concentrations in alfalfa and lentil sprouts. *Epi*-inositol was found in higher concentrations only in lentil sprouts and in small amounts in clover, while mung bean sprouts contained *scyllo*-inositol. Data on the presence and content of cyclitols in the sprouts of the above-mentioned species have not been published so far, except for mung bean [54]. According to these data, the DCI content in sprouts of different mung bean species ranged from 0.43 to 5.79 mg/g dry weight and increased during germination [54]. DCI and d-pinitol are important cyclitols because both show promising effects in reducing the disorders associated with type 2 diabetes [54,55,56,57]. D-pinitol has been suggested to have therapeutic potential due to its anti-inflammatory and anticancer activity [58]. In turn, *epi*-inositol is an antidepressant agent that also shows an antiepileptic effect [59], while *scyllo*-inositol may be helpful in the treatment of Alzheimer’s disease [60]. Therefore, the information obtained during this study regarding the presence and content of the above-mentioned cyclitols in sprouts could be important.

Sprouts are a site of intense metabolism, resulting in a high content of free amino acids (FAAs) needed for protein biosynthesis and plant development. In turn, storage proteins contained in seeds undergo proteolysis during germination, resulting in an increased content of FAAs [61]. The results of our analyses indicate that *Fabaceae* sprouts have a significantly higher content of free proteinogenic FAAs than the sprouts of *Brassicaceae* species and sunflower sprouts (Table 1). Furthermore, *Fabaceae* sprouts had much higher essential FAA contents than *Brassicaceae* sprouts or sunflower sprouts. Among FAAs, *Fabaceae* sprouts contained the highest asparagine content, especially in mung bean, clover, and alfalfa (Table S5). These results confirm previously published data [8,62]. Earlier, Rozan et al. [62] reported that in 4-day-old sprouts of five lentil species, the quantitatively main free amino acid was asparagine, and among the tested *Lens* species, the lowest content was found in *L. culinaris* and the highest in *L. odemensis*.

During the germination process, in addition to the hydrolysis of stored proteins to free amino acids, glutamate is decarboxylated to γ-aminobutyric acid (GABA) [63,64]. Generally, sprouting increases the GABA content in many species: adzuki beans, red beans, lentils, lupins, sesame, soybeans, peas, brown rice, buckwheat, wheat, and oats [65]. During our study, the GABA content ranged from 0.05 to 0.25 mg/g DW in sprouts of *Brassicaceae* species and from 0.22 to 0.55 mg/g DW in sprouts of *Fabaceae* species, while fresh sunflower sprouts contained 0.38 mg GABA/g DW (Tables S3–S5). Kuo et al. [61] have reported that GABA was not present in dry seeds, but its content increased significantly during germination. The presence of GABA in food is important because it is an important neurotransmitter in the mammalian nervous system, and it can also increase insulin secretion by the pancreas, regulate blood pressure and heart rate, and relieve pain and anxiety [66,67].

In addition to GABA, the presence of beta-alanine in broccoli and *Fabaceae* sprouts and hydroxyproline in broccoli, kale, sunflower, and mung bean sprouts were also demonstrated in present study (Tables S3–S5). Martínez-Villaluenga et al. [68] showed that longer germination time causes a significant increase in β-alanine content in soybean and lupin (*Lupinus angustifolius* L.) sprouts. β-Alanine is part of pantothenate, which is part of the universal carbon transport compounds, like coenzyme A, and proteins that transport carbon within the cell in all organisms, including plants [69].

Malic and citric acids were found in the sprouts of all species (Tables S3–S5). The highest contents of these acids were found in mung bean sprouts. Different results of organic acid analyses in sprouts of several species were reported by Wojdyło et al. [8]. According to these authors, the main organic acid in radish, lentil, broccoli, sunflower, and mung bean sprouts was oxalic acid. In our study, the presence of small levels of oxalic acid was confirmed in sprouts of all species, but its contents were low. In turn, the data published by Vale et al. [31] showed that broccoli sprouts contained the highest citric acid content, which was many times higher than the content of oxalic and malic acids. For comparison, in our study, the level of citric acid in broccoli sprouts was 2.33 mg/g DW, and malic acid was 4.99 mg/g DW (Table 1). The differences between these data are likely influenced by the conditions in which the sprouts were obtained or due to the broccoli varieties used.

### 3.2. Changes in Metabolic Profile During Cold Storage of Sprouts

To prevent quality deterioration and extend the shelf life of food, cold storage is usually used [30,47,70,71,72]. This method is widely used because refrigerators are found in almost every home today. In radish sprouts, the visual quality was better when they were stored at 4 °C due to the lower constant respiration rate [73,74]. For instance, storage temperatures of 4–5 °C have been recommended to avoid losses of bioactive compounds, and to maintain shelf life up to 14 days in broccoli and radish sprouts [17].

There is little data in the available scientific literature on the effect of storing sprouts under refrigerated conditions on the content of particular carbohydrates. However, the accumulation of carbohydrates in plant tissues in response to cold stress seems to be a common phenomenon [75,76,77]. Our research indicates that the effect of cold storage on the content of carbohydrates in sprouts depends on their species (Tables S3–S5). After cold storage, the fructose content decreased in kale, alfalfa, lentil, and mung bean sprouts, while glucose levels decreased in broccoli, kale, alfalfa, and lentil sprouts. These storage conditions increased the sucrose content in broccoli, kale, radish, and mung bean sprouts, as well as the raffinose content in broccoli, kale, radish, and clover. In the case of lentil sprouts, cold storage conditions had no effect on sucrose or raffinose contents. In turn, in alfalfa sprouts, the content of monosaccharides, sucrose, and raffinose decreased after cold storage. Moreover, cold storage conditions enhanced content *myo*-inositol in *Brassicaceae* and sunflower sprouts, but not in legume sprouts. In our recently published paper, we reported that cold stress increased sucrose and raffinose concentrations in the cotyledons and roots of fenugreek sprouts, but it did not affect the *myo*-inositol content in the cotyledons and hypocotyl and only slightly decreased their levels in the roots [78]. This suggests that the lack of effect of cold stress on *myo*-inositol levels is a common phenomenon in *Fabaceae* sprouts.

An amino acid that plays a role in plant stress response is proline [79]. In our study, proline content generally decreased or remained unchanged following cold storage. At the same time, significant decreases in the proline content of broccoli sprouts and kale were accompanied by increases in hydroxyproline content. According to present results, free asparagine was quantitatively the main amino acid in lentil sprouts, confirming previous data [62]. In sprouts of most species (except kale, clover, and mung bean), storage conditions increased the content of asparagine. The increase in proline and asparagine was shown in wheat seedlings in response to cold stress [80,81,82]. Cold stress caused an increase in asparagine content in *Pinus halepensis* sprouts [82]. The accumulation of asparagine in response to abiotic stress may be related to the way nitrogen is stored when protein synthesis is inhibited by stress conditions [83].

Cold storage caused a decrease in the citric acid content in sprouts of all *Fabaceae* species, whereas in broccoli sprouts and kale, the level of this acid increased. The available literature does not provide any data on the effect of cold on the content of malic and citric acids.

### 3.3. Changes in Metabolic Profile During Mild Drying of Sprouts

The quality of dried sprouts is influenced by the drying conditions and method used, which must be selected to fully preserve the nutritional value of the sprouts [32]. In plants subjected to drought stress, the accumulation of RFOs is a common phenomenon and plays an important role in the protection and stabilization of proteins [33]. Cyclitols and their galactosides may also accumulate in the tissues of some plant species in response to desiccation [51,52]. The biosynthesis and accumulation of galactinol and raffinose results from the induction of galactinol synthase and raffinose synthase gene expression [46,51].

In the present study, the process of mild drying resulted in a significant increase in sucrose content (Tables S3–S5). A particularly large increase in sucrose content following drying occurred in the sprouts of *Brassicaceae* species, while a smaller increase was observed in *Fabaceae* sprouts. The increase in sucrose content in response to drying was accompanied by the accumulation of raffinose in the sprouts of all species except mung bean. Furthermore, the contents of glucose and fructose decreased significantly because they were likely used for sucrose biosynthesis. It seems that the formation of sucrose as a result of a mild drying process is a common process, independent of the species of sprouts studied [51,52,79]. In the case of galactinol, a slight increase was also observed as a result of the drying conditions used but only in *Brassicaceae* sprouts. The slight reaction in galactinol content in response to drying conditions probably indicates its use in raffinose biosynthesis. Previously, it was shown that in buckwheat sprouts, raffinose underwent far greater changes in the hypocotyl than in the cotyledons and roots following drying [51]. This may indicate tissue differences in the functioning of protective systems against dehydration stress. Raffinose biosynthesis occurs by the action of raffinose synthase, which catalyzes the transfer of a galactosyl fragment from galactinol to sucrose [84,85,86]. Both galactinol and raffinose also have the ability to remove hydroxyl radicals and protect plant cells from oxidative damage [76]. Additionally, in plants, raffinose plays a role in carbon transport and storage [87].

Important information is the presence of free protein amino acids in sprouts (Table S2). Interestingly, the applied drying process conditions increased their total content in the sprouts of all tested species. A particularly large three-fold increase was recorded in radish sprouts, while in the sprouts of other species, this was much lower. The total level of essential amino acids also increased in dried sprouts compared to fresh in most species, with the exception of mung bean and broccoli. This information has not been published before. Among the free protein amino acids, proline in particular increased significantly after drying (Tables S3–S5). In dried broccoli, radish, kale, lentil, and clover sprouts, the proline content was approximately 3–4 times higher than in fresh ones, while in the remaining species, it ranged from 40% in mung bean to 200% in sunflower.

It is well-known that plant tissues exposed to abiotic stress conditions will accumulate amino acids [88,89]. Our results confirm this as the content of asparagine, proline, and most other amino acids increased in the sprouts of all tested species. GABA content increased after drying in sprouts of all species, except lentil, which is important information due to its potential pharmacological role [66].

Drying sunflower and legume sprouts reduced malic acid levels, while a slight increase was observed in cruciferous sprouts. In the case of citric acid, in almost all species, its content decreased or remained unchanged after drying the sprouts. There is limited data in the available literature regarding the contents and roles of these acids during drought stress. High levels of malic acid are generally believed to increase drought tolerance in various plant species, such as cotton, and tropical grasses [90]. However, according to Khosravi-nejad et al. [91], drought stress caused a decrease in the concentration of malic acid, pyruvic acid, and citric acid in wheat cultivars.

## 4. Materials and Methods

### 4.1. Plant Material

Sprouts were purchased in October 2023 from local food markets: Auchan and Lidl in Olsztyn (Poland). Sprouts of *Brassicaceae* species (broccoli, kale, radish), alfalfa, and mung bean were produced by Food of Life Co. (Warsaw, Poland), whereas sprouts of sunflower and clover were produced by Uniflora Co. (Częstochowa, Poland). Lentil seeds were purchased from W. Legutko Breeding and Seed Company Co. (Jutrosin, Poland) and germinated for 7 days at 22 °C in a 12/12 h photoperiod in a climatic chamber (ILW 115-T STD, POL-EKO APARATURA (Wodzisław Śląski, Poland). Fresh sprouts (divided into 3 batches, 10–15 g each) were frozen in liquid nitrogen and then freeze-dried (Alpha 1–2 LD plus, Christ, Osterode, Germany). Part of the sprouts in the original manufacturer’s plastic bag, weighing 50 g, were stored in a refrigerator at +4 − +6 °C for 7 days, after which the sprouts were frozen in three separate batches of 10–12 g each, and then freeze-dried. The relative air humidity during low-temperature storage of sprouts in their original packaging was 70–80%. Another batch of sprouts was dried for 7–10 days in ambient laboratory conditions, at a temperature of 22 °C, with a relative air humidity of 40–50% and free air circulation, until the water content reached a level less than 8% of the fresh weight.

### 4.2. Extraction of Polar Metabolites

Dried sprouts were pulverized in a mixer mill (MM200, Retsch, Haan-Gruiten, Germany), and samples of flour (40–45 mg) were prepared in 2 mL round-bottom microcentrifuge tubes before adding 900 μL of 50% methanol containing 100 μg of ribitol (internal standard). The samples were heated at 70 °C for 30 min (with continuous shaking, 500 rpm) and centrifuged (20,000× *g* at 4 °C, 20 min). Supernatant (600 μL) was transferred to 1.5 mL Eppendorf tubes and non-polar compounds were removed by extraction with 400 μL of cold chloroform. The tubes were shaken (1350 rpm, 15 min), and after centrifugation (20,000× *g* at 4 °C, 10 min), 200 μL of the supernatant was concentrated in 2 mL chromatographic vials (containing 350 μL glass inserts) in a vacuum rotary evaporator (JW Electronic, Warsaw, Poland) until dry. The vials were closed in a neutral gas (nitrogen) and stored in desiccators, above silica gel.

### 4.3. Gas Chromatography Analyses

Dry samples were derivatized in two steps according to the method described by Lisec et al. [92]. Firstly, after the addition of 40 μL of *O*-methoxamine hydrochloride (concentration 20 mg/mL in pyridine), the sample was heated (37 °C) for 75 min with continuous shaking at 500 rpm. Next, 160 μL of a MSTFA mixture (*N*-methyl-*N*-trimethylsilyl-trifluoroacetamide in pyridine, 1:1, *v*/*v*) was added and the mixture was heated at 70 °C for 30 min.

After cooling the obtained TMS derivatives were separated in a ZEBRON ZB5-MSi Guardian columns (length of 30 m, ø 0.25 mm, and 0.25 μm film thickness (5% phenyl-95% dimethyl-polysiloxane) (Phenomenex, Torrance, CA, USA) in two gas chromatographs: GC-2010 (Shimadzu, Kyoto, Japan) equipped with a flame ionization detector (FID); and GC-2010 coupled with a quadrupole mass spectrometry (MS) analyzer (GCMS-QP2010 Plus, Shimadzu, Kyoto, Japan). Metabolites were identified by comparison of retention time (*t_R_*), relative retention time (RRT), and mass spectra of original standards derived from Sigma-Aldrich (Burlington, MA, USA) and from the NIST 05 library (National Institute of Standards and Technology, NIST, USA). The same parameters for chromatographic separation were applied in GC-FID and GC–MS analyses. The analyses in GC-FID were used for identification and quantification of metabolites, while GC-MS was used to confirm the proper identification of metabolites. The column temperature increase was programmed at a rate optimized for best metabolite separation, as previously described [93]. Data were collected and analyzed using GC–MS Solution software ver. 2.6 (Shimadzu, Kyoto, Japan). The concentration of identified polar metabolites was calculated according to the method described previously [93,94].

### 4.4. Statistics

The obtained results (means of three independent replicates) were subjected to one-way ANOVA and a post hoc test (Tukey) using Statistica software (version 12.0; StatSoft, Tulsa, OK, USA). Graphs were prepared using GraphPad Prism (version 8.0; GraphPad Software, San Diego, CA, USA). Principal component analysis (PCA) was performed in the COVAIN program [94], using the MATLAB software (version 2013a, Math Works, Natick, MA, USA).

## 5. Conclusions

This study compared the polar compound profiles in commercially available sprouts of three *Brassicaceae* species, four *Fabaceae* species, and sunflower. In addition, the influences of the cold storage process and the mild drying process in ambient conditions on the composition of the tested sprouts were assessed. *Brassicaceae* and sunflower sprouts contained higher contents of monosaccharides than *Fabaceae* sprouts, except for mung bean. Sprouts of all tested species contained *myo*-inositol, whereas its methyl derivative, d-pinitol, was found in measurable amounts only in alfalfa, clover, and lentil sprouts. Alfalfa and lentil sprouts contained small amounts of d-*chiro*-inositol, while *epi*-inositol was found only in lentil and clover sprouts, and *scyllo*-inositol in mung bean sprouts. Furthermore, *Fabaceae* sprouts had much higher contents of essential free amino acids and asparagine than *Brassicaceae* or sunflower sprouts.

Applied cold storage conditions led to a decrease in fructose content in kale, alfalfa, lentil, and mung bean sprouts, as well as glucose in broccoli, kale, alfalfa, and lentil sprouts. These storage conditions increased the sucrose content in broccoli, kale, radish and mung bean sprouts, and the raffinose content in broccoli, kale, radish, and clover. The cyclitol content in sprouts remained almost unchanged, both during cold storage and the drying process. Proline content generally decreased or remained unchanged following cold storage of sprouts, while asparagine levels increased in most species.

The process of mild drying resulted in a dramatic increase in sucrose content in sprouts of all the investigated species, as well as an increase in raffinose content (except for mung bean). This process also decreased glucose and fructose contents in sprouts of all species and increased total content of free amino acids in the sprouts of all tested species. The total level of essential amino acids also increased in dried sprouts compared to fresh ones in most species, with the exception of mung bean and broccoli. The process of mild drying highly increased the proline content in all investigated sprout species, while GABA content increased in sprouts of all species except lentils.

In general, changes in polar compound profiles in the sprouts of the examined species due to cold storage and drying processes conducted under mild conditions were minor. However, the reduction in the content of monosaccharides and the increase in the content of exogenous amino acids, as well as proline and GABA in dried sprouts, is new information. Furthermore, it is important to note that both storage at low temperatures and drying of sprouts had a small effect on the content of *myo*-inositol, d-pinitol, and *scyllo*-inositol. Future comparative studies of different sprout species should take into account the content of secondary metabolites.

## Figures and Tables

**Table 1:** The concentration of polar metabolites (mg/g DW ± SD) in sprouts of *Brassicaceae* (broccoli, kale, and radish), *Asteraceae* (sunflower), and *Fabaceae* (alfalfa, clover, lentil, and mung bean). Results marked with the same letters (compared in rows) indicate no significant differences (*p* < 0.05) after one-way ANOVA and Tukey’s test. Abbreviations: bdl—below the detection limit, 0.01 mg/g DW.

Metabolite	*Brassicaceae*			*Asteraceae*	*Fabaceae*			
	Broccoli	Kale	Radish	Sunflower	Alfalfa	Clover	**Lentil**	**Mung Bean**
Citric acid	2.33 ± 0.19 ^D^	2.71 ± 0.60 ^CD^	3.59 ± 0.30 ^BC^	1.63 ± 1.10 ^D^	4.74 ± 0.44 ^B^	2.25 ± 0.06 ^D^	2.92 ± 0.12 ^CD^	17.88 ± 0.41 ^A^
Erythronic acid	0.15 ± 0.01 ^A^	0.15 ± 0.01 ^A^	bdl	bdl	0.07 ± 0.02 ^BC^	0.10 ± 0.01 ^B^	0.04 ± 0.01 ^C^	0.06 ± 0.01 ^BC^
Fumaric acid	bdl	0.08 ± 0.01 ^C^	0.03 ± 0.01 ^C^	0.81 ± 0.12 ^B^	bdl	1.17 ± 0.05 ^A^	bdl	bdl
Lactic acid	0.18 ± 0.00 ^A^	0.03 ± 0.00 ^DE^	0.02 ± 0.01 ^E^	0.04 ± 0.02 ^CD^	0.11 ± 0.00 ^B^	0.06 ± 0.00 ^C^	0.04 ± 0.00 ^CDE^	0.05 ± 0.00 ^CD^
Malic acid	4.99 ± 0.04 ^C^	6.28 ± 0.34 ^B^	3.07 ± 0.12 ^D^	3.73 ± 0.23 ^D^	2.42 ± 0.07 ^E^	2.02 ± 0.04 ^E^	2.21 ± 0.07 ^E^	11.46 ± 0.43 ^A^
Oxalic acid	0.12 ± 0.02 ^BCD^	0.09 ± 0.01 ^CDE^	0.04 ± 0.01 ^F^	0.05 ± 0.01 ^EF^	0.18 ± 0.02 ^A^	0.14 ± 0.00 ^ABC^	0.15 ± 0.02 ^AB^	0.07 ± 0.03 ^DEF^
Propanoic acid	0.64 ± 0.01 ^B^	0.73 ± 0.05 ^B^	0.67 ± 0.03 ^B^	0.07 ± 0.01 ^C^	bdl	1.81 ± 0.08 ^A^	bdl	0.08 ± 0.01 ^C^
Succinic acid	1.99 ± 0.03 ^A^	1.32 ± 0.08 ^B^	bdl	bdl	bdl	bdl	bdl	0.76 ± 0.05 ^C^
Glycerol	2.52 ± 0.07 ^D^	1.96 ± 0.08 ^E^	3.81 ± 0.18 ^C^	6.94 ± 0.25 ^A^	0.66 ± 0.07 ^G^	0.42 ± 0.02 ^G^	4.72 ± 0.11 ^B^	1.15 ± 0.14 ^F^
Phosphoric acid	3.29 ± 0.13 ^D^	2.57 ± 0.17 ^E^	2.24 ± 0.15 ^E^	5.22 ± 0.37 ^BC^	7.12 ± 0.26 ^A^	4.94 ± 0.03 ^BC^	4.72 ± 0.11 ^C^	5.45 ± 0.18 ^B^
Alanine	0.22 ± 0.03 ^E^	0.30 ± 0.02 ^DE^	0.18 ± 0.03 ^E^	0.35 ± 0.04 ^D^	1.43 ± 0.08 ^A^	0.67 ± 0.02 ^C^	0.99 ± 0.05 ^B^	0.73 ± 0.05 ^C^
Asparagine	0.28 ± 0.00 ^D^	0.51 ± 0.03 ^D^	0.03 ± 0.02 ^D^	1.41 ± 0.20 ^D^	27.25 ± 1.34 ^A^	25.68 ± 1.35 ^A^	7.80 ± 0.22 ^C^	21.63 ± 1.48 ^B^
Aspartic acid	0.17 ± 0.00 ^E^	0.24 ± 0.01 ^DE^	0.35 ± 0.05 ^C^	0.30 ± 0.02 ^CD^	0.95 ± 0.08 ^A^	0.60 ± 0.04 ^B^	0.36 ± 0.01 ^C^	0.86 ± 0.00 ^A^
Glutamic acid	0.76 ± 0.05 ^DE^	0.32 ± 0.12 ^EF^	0.10 ± 0.01 ^F^	2.25 ± 0.57 ^AB^	2.28 ± 0.11 ^A^	1.65 ± 0.09 ^BC^	1.34 ± 0.12 ^CD^	0.35 ± 0.05 ^EF^
Glycine	0.51 ± 0.04 ^D^	0.55 ± 0.02 ^D^	0.12 ± 0.04 ^E^	0.54 ± 0.03 ^D^	1.36 ± 0.08 ^A^	1.07 ± 0.05 ^B^	0.77 ± 0.03 ^C^	0.25 ± 0.03 ^E^
Leucine	0.37 ± 0.02 ^B^	0.46 ± 0.02 ^B^	0.15 ± 0.01 ^C^	0.44 ± 0.01 ^B^	0.23 ± 0.02 ^C^	0.13 ± 0.01 ^C^	0.15 ± 0.00 ^C^	0.93 ± 0.13 ^A^
Lysine	1.19 ± 0.09 ^B^	1.15 ± 0.05 ^B^	0.47 ± 0.09 ^C^	bdl	0.71 ± 0.03 ^BC^	0.20 ± 0.01 ^C^	bdl	7.17 ± 0.49 ^A^
Methionine	0.24 ± 0.01 ^A^	0.23 ± 0.01 ^A^	0.05 ± 0.01 ^B^	0.20 ± 0.01 ^A^	0.22 ± 0.08 ^A^	0.09 ± 0.01 ^B^	0.05 ± 0.00 ^B^	0.20 ± 0.05 ^A^
Phenylalanine	0.33 ± 0.03 ^DE^	0.47 ± 0.02 ^D^	bdl	0.18 ± 0.01 ^E^	2.03 ± 0.11 ^B^	2.03 ± 0.03 ^B^	0.95 ± 0.01 ^C^	4.20 ± 0.14 ^A^
Proline	1.30 ± 0.05 ^BC^	1.43 ± 0.07 ^B^	0.86 ± 0.04 ^D^	1.65 ± 0.03 ^B^	1.40 ± 0.06 ^B^	0.77 ± 0.01 ^D^	1.01 ± 0.04 ^CD^	2.56 ± 0.34 ^A^
Serine	0.72 ± 0.08 ^E^	1.02 ± 0.05 ^D^	0.65 ± 0.03 ^E^	0.84 ± 0.09 ^DE^	5.00 ± 0.24 ^A^	2.92 ± 0.04 ^B^	2.24 ± 0.12 ^C^	2.28 ± 0.01 ^C^
Threonine	1.18 ± 0.10 ^D^	1.40 ± 0.06 ^CD^	0.64 ± 0.03 ^E^	0.72 ± 0.04 ^E^	3.37 ± 0.17 ^A^	2.43 ± 0.12 ^B^	1.51 ± 0.05 ^C^	3.57 ± 0.15 ^A^
Tryptophan	bdl	0.20 ± 0.00 ^D^	0.22 ± 0.02 ^D^	0.46 ± 0.13 ^C^	1.36 ± 0.09 ^A^	0.73 ± 0.09 ^B^	0.30 ± 0.02 ^CD^	bdl
Tyrosine	0.32 ± 0.04 ^B^	0.45 ± 0.03 ^B^	bdl	0.24 ± 0.00 ^B^	0.71 ± 0.02 ^B^	0.33 ± 0.01 ^B^	0.39 ± 0.01 ^B^	3.35 ± 0.53 ^A^
Valine	1.06 ± 0.03 ^E^	1.34 ± 0.06 ^D^	0.59 ± 0.03 ^F^	0.89 ± 0.04 ^E^	3.02 ± 0.16 ^B^	1.73 ± 0.04 ^C^	1.80 ± 0.02 ^C^	4.90 ± 0.13 ^A^
β-alanine	0.04 ± 0.01 ^D^	bdl	bdl	bdl	0.45 ± 0.02 ^A^	0.17 ± 0.01 ^C^	0.06 ± 0.00 ^D^	0.32 ± 0.01 ^B^
GABA	0.29 ± 0.02 ^D^	0.21 ± 0.00 ^E^	0.05 ± 0.01 ^G^	0.38 ± 0.02 ^C^	0.44 ± 0.04 ^B^	0.22 ± 0.03 ^E^	0.55 ± 0.02 ^A^	0.13 ± 0.01 ^F^
Homoserine	bdl	bdl	bdl	bdl	3.59 ± 0.32 ^A^	0.16 ± 0.03 ^B^	0.03 ± 0.01 ^B^	bdl
Hydroxyproline	1.51 ± 0.17 ^B^	2.26 ± 0.29 ^A^	bdl	0.76 ± 0.24 ^C^	bdl	bdl	bdl	0.42 ± 0.18 ^C^
Ciceritol	bdl	bdl	bdl	bdl	0.23 ± 0.05 ^A^	0.20 ± 0.05 ^A^	0.08 ± 0.00 ^B^	0.06 ± 0.01 ^B^
Fructose	30.85 ± 1.46 ^B^	22.35 ± 0.97 ^C^	17.91 ± 0.88 ^D^	43.08 ± 1.35 ^A^	9.10 ± 0.91 ^E^	0.62 ± 0.07 ^F^	0.76 ± 0.05 ^F^	44.57 ± 0.26 ^A^
Galactinol	0.04 ± 0.01 ^BC^	0.07 ± 0.01 ^BC^	0.02 ± 0.00 ^C^	bdl	0.51 ± 0.03 ^A^	0.04 ± 0.01 ^BC^	bdl	0.08 ± 0.01 ^B^
Galactose	0.88 ± 0.51 ^B^	bdl	4.54 ± 0.22 ^A^	3.32 ± 2.14 ^AB^	1.81 ± 0.01 ^B^	bdl	0.93 ± 0.03 ^B^	bdl
Gluconic acid	0.09 ± 0.02 ^B^	0.24 ± 0.02 ^B^	bdl	bdl	0.65 ± 0.11 ^A^	0.69 ± 0.08 ^A^	0.14 ± 0.01 ^B^	0.23 ± 0.02 ^B^
Glucose	44.76 ± 2.09 ^B^	35.76 ± 1.52 ^C^	19.74 ± 0.91 ^D^	71.46 ± 3.21 ^A^	3.56 ± 0.45 ^E^	1.19 ± 0.01 ^E^	2.67 ± 0.28 ^E^	20.08 ± 0.69 ^D^
Maltose	bdl	bdl	bdl	bdl	0.06 ± 0.01 ^B^	bdl	0.16 ± 0.03 ^A^	bdl
Raffinose	0.06 ± 0.03 ^CD^	0.11 ± 0.02 ^BC^	0.08 ± 0.05 ^B^	0.10 ± 0.03 ^BC^	0.37 ± 0.02 ^A^	bdl	0.03 ± 0.01 ^D^	0.09 ± 0.01 ^BCD^
Sucrose	13.57 ± 0.17 ^C^	14.91 ± 0.99 ^C^	11.84 ± 0.44 ^C^	33.51 ± 2.62 ^B^	4.15 ± 0.11 ^D^	2.68 ± 0.12 ^D^	31.32 ± 1.42 ^B^	60.88 ± 2.66 ^A^

**Table 2:** The concentration (mg/g DW ± SD) of cyclitols in sprouts before (initial, control) and after cold storage or drying. Results marked with the same letters (compared separately in rows) indicate no significant differences (*p* < 0.05) after ANOVA and Tukey’s test. Abbreviations: bdl—below the detection limit, 0.01 mg/g DW.

Sprouts	Initial (Control)	Cold Storage	Drying
	*Myo*-Inositol		
Broccoli	1.62 ± 0.02 ^B^	2.15 ± 0.04 ^A^	2.13 ± 0.07 ^A^
Kale	1.31 ± 0.08 ^B^	1.56 ± 0.05 ^A^	1.60 ± 0.02 ^A^
Radish	0.87 ± 0.07 ^B^	1.33 ± 0.10 ^A^	1.41 ± 0.09 ^A^
Sunflower	2.09 ± 0.12 ^B^	2.42 ± 0.07 ^A^	2.57 ± 0.13 ^A^
Alfalfa	1.19 ± 0.04 ^A^	1.00 ± 0.04 ^B^	0.98 ± 0.03 ^B^
Clover	0.99 ± 0.02 ^A^	1.07 ± 0.09 ^A^	0.71 ± 0.02 ^B^
Lentil	2.83 ± 0.11 ^A^	2.82 ± 0.06 ^A^	2.86 ± 0.20 ^A^
Mung bean	2.31 ± 0.06 ^A^	1.15 ± 0.07 ^C^	1.97 ± 0.06 ^B^
	d-Pinitol		
Alfalfa	4.67 ± 0.07 ^A^	4.82 ± 0.25 ^A^	4.78 ± 0.08 ^A^
Clover	3.56 ± 0.18 ^A^	2.49 ± 0.21 ^B^	3.98 ± 0.16 ^A^
Lentil	7.46 ± 0.27 ^B^	6.70 ± 0.11 ^B^	7.74 ± 0.42 ^A^
Mung bean	bdl	bdl	bdl
	d-*chiro*-Inositol		
Alfalfa	0.46 ± 0.07 ^B^	0.62 ± 0.06 ^A^	0.60 ± 0.02 ^A^
Clover	bdl	bdl	bdl
Lentil	0.03 ± 0.01 ^A^	0.04 ± 0.01 ^A^	0.05 ± 0.01 ^A^
Mung bean	bdl	bdl	bdl
	*Epi*-Inositol		
Alfalfa	bdl	bdl	bdl
Clover	0.11 ± 0.02 ^A^	0.05 ± 0.02 ^A^	0.11 ± 0.02 ^A^
Lentil	2.83 ± 0.11 ^A^	2.82 ± 0.06 ^A^	2.86 ± 0.20 ^A^
Mung bean	bdl	bdl	bdl
	*Scyllo*-Inositol		
Alfalfa	bdl	bdl	bdl
Clover	bdl	bdl	bdl
Lentil	bdl	bdl	bdl
Mung bean	0.70 ± 0.05 ^A^	0.27 ± 0.01 ^B^	0.66 ± 0.01 ^A^

## Data Availability

All the data of this study are included in this article and the Supplementary Materials.

## Supplementary Materials

The following supporting information can be downloaded at: https://www.mdpi.com/article/10.3390/molecules31142442/s1, Plate S1. Pictures of fresh and dried sprouts of Brassicaceae (broccoli, kale, radish), sunflower, and Fabaceae (alfalfa, clover, lentil, and mung bean). Figure S1. GC chromatograms of TMS derivatives of polar metabolites extracted from control sprouts of *Brassicaceae* (broccoli, kale, radish) and sunflower. Figure S2. GC chromatograms of TMS derivatives of polar metabolites extracted from control sprouts of *Fabaceae* (alfalfa, clover, lentil, and mung bean). Figure S3. PCA scores and loading plots of the soluble carbohydrate profiles (A,B) or proteinogenic amino acid profiles (C,D) of control sprouts of eight edible species. Figure S4. PCA scores (A,C,E,G) and loading plots (B,D,F,H) of the polar metabolic profiles of broccoli (A,B), kale (C,D), radish (E,F), and sunflower (G,H) sprouts. Symbols: circles—control, squares—after 7 days of cold stress (CS), and triangles—after 7–10 days of drying (DS). Figure S5. PCA scores (A,C,E,G) and loading plots (B,D,F,H) of the polar metabolic profiles of alfalfa (A,B), clover (C,D), lentil (E,F), and mung bean (G,H) sprouts. Symbols: circles—control, squares—after 7 days of cold stress (CS), and triangles—after 7–10 days of drying (DS). Table S1. Retention times (RTs) and relative retention times (RRTs) of identified polar metabolites. Table S2. The concentration (in mg/g DW) of total polar metabolites (TPMs), including total soluble carbohydrates (TSCs); total amino acids (TAAs); which include total essential amino acids (TEAAs) and total non-proteinogenic amino acids (TNPAAs); total organic acids (TOAs), and total other compounds (TOCs), in sprouts before (initial, control) and after cold storage or after drying. Results marked with the same letters (compared separately in rows) indicate no significant differences (*p* < 0.05) after ANOVA and Tukey’s test. Table S3. The concentration (mg/g DW ± SD) of polar metabolites in sprouts of *Brassicaceae* (broccoli, kale, and radish) before (control) and after cold storage or after drying. Results marked with the same letters (compared in rows, separately for each species) indicate no significant differences (*p* < 0.05) after ANOVA and Tukey’s test. Table S4. The concentration (mg/g DW ± SD) of polar metabolites before (control) and after cold storage or after drying of sunflower sprouts. Results marked with the same letters (compared in rows) indicate no significant differences (*p* < 0.05) after ANOVA and Tukey’s test. Table S5. The concentration (mg/g DW ± SD) of polar metabolites in sprouts of legumes (alfalfa, clover, lentil, and mung bean) before (control) and after cold storage or after drying. Results marked with the same letters (compared in rows, separately for each species) indicate no significant differences (*p* < 0.05) after ANOVA and Tukey’s test.

## Abbreviations

The following abbreviations are used in this manuscript:

SD	Standard Deviation
bdl	Below the Detection Limit
DW	Dry Weight
PCA	Principal Component Analysis
GABA	γ-Amino Butyric Acid
GC-MS	Gas Chromatography Coupled to Mass Spectrometry
RFO	Raffinose Family Oligosaccharide
