# Supplementary material for: Cold Storage and Drying Alter Polar Metabolite Profiles in Commercial Sprouts of Eight Plant Species
Source: Molecules. 2026 Jul 12;31(14):2442. doi: 10.3390/molecules31142442 (PMC13415856; doi:10.3390/molecules31142442)
Supplement: Supplementary file 1 [file molecules-31-02442-s001.zip › Supplementary Figures.pdf]

Supplementary materials

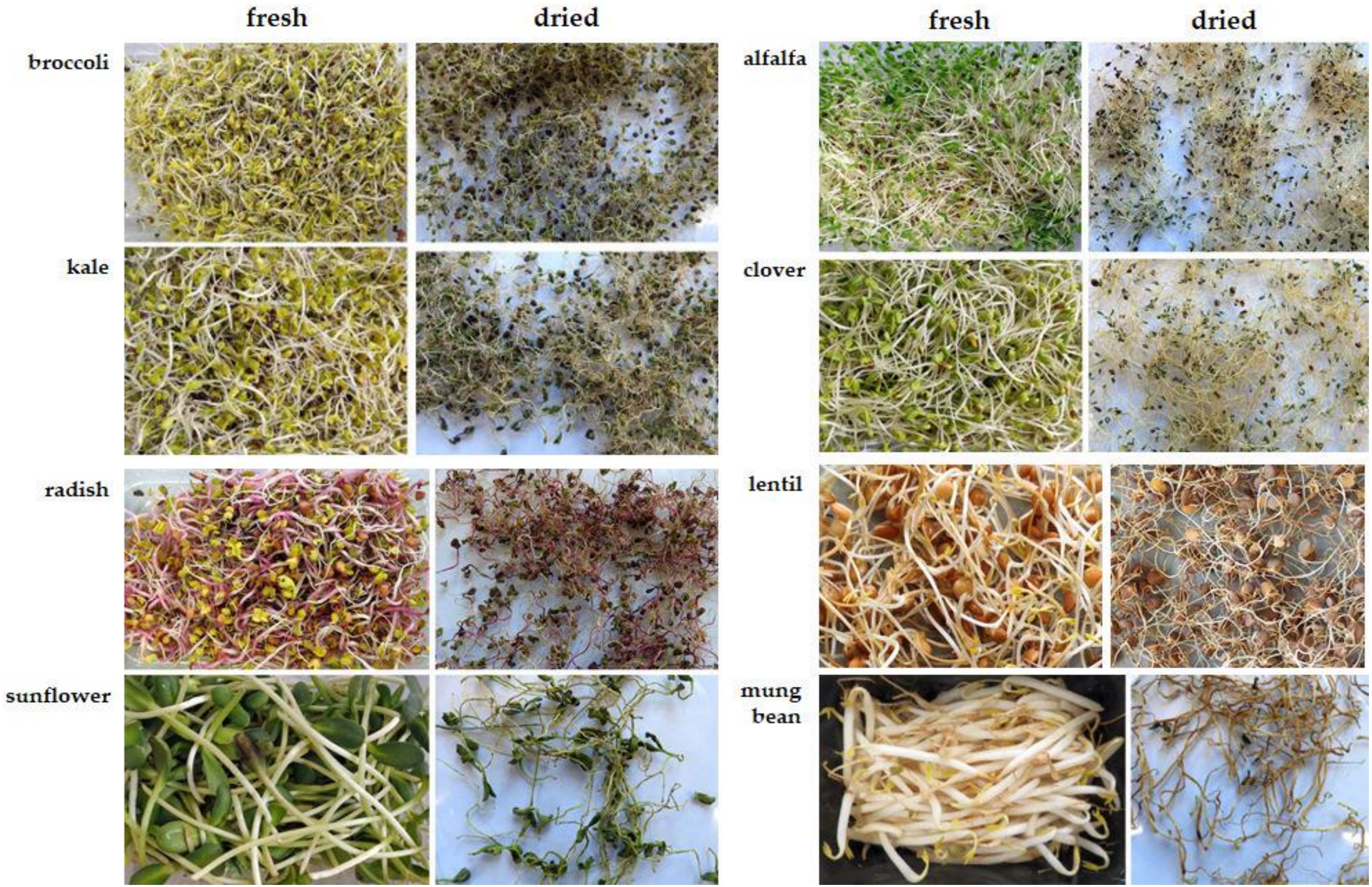

Plate S1. The pictures of fresh and dried sprouts of Brassicaceae (broccoli, kale, radish), sunflower and Fabaceae (alfalfa, clover, lentil and mung bean).

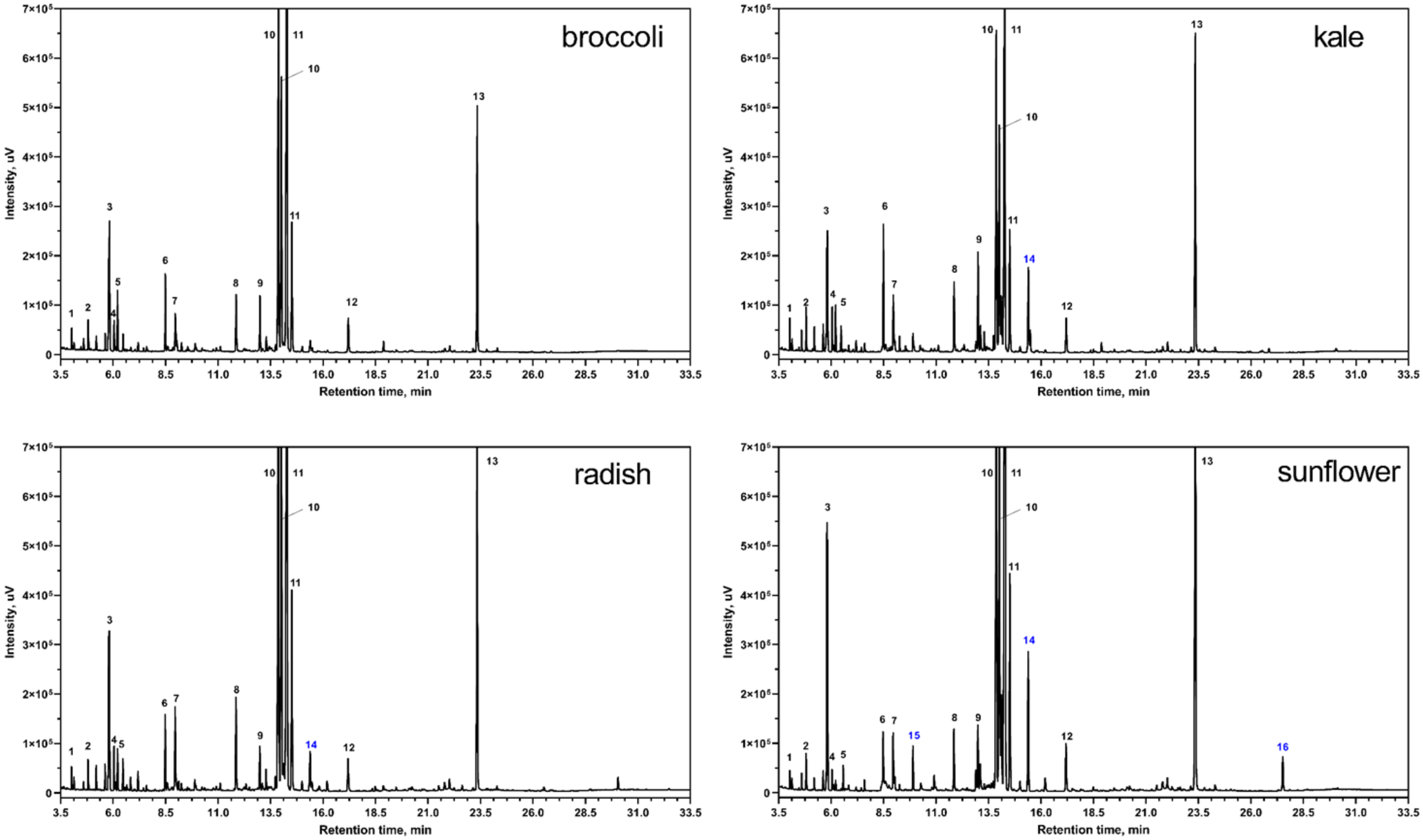

Abbreviations: 1 - valine; 2 - proline; 3 - phosphoric acid; 4 - threonine; 5 - succinic acid; 6 - malic acid; 7 - hyroxyproline; 8 - ISDT (xylitol); 9 - citric acid; 10 - fructose; 11 - glucose; 12 - *myo*-inositol; 13 - sucrose; 14 - UNK2; 15 - asparagine; 16 - chlorogenic acid.

Figure S1. The GC-chromatograms of TMS-derivatives of polar metabolites extracted from control sprouts of Brassicaceae (broccoli, kale, radish) and sunflower.

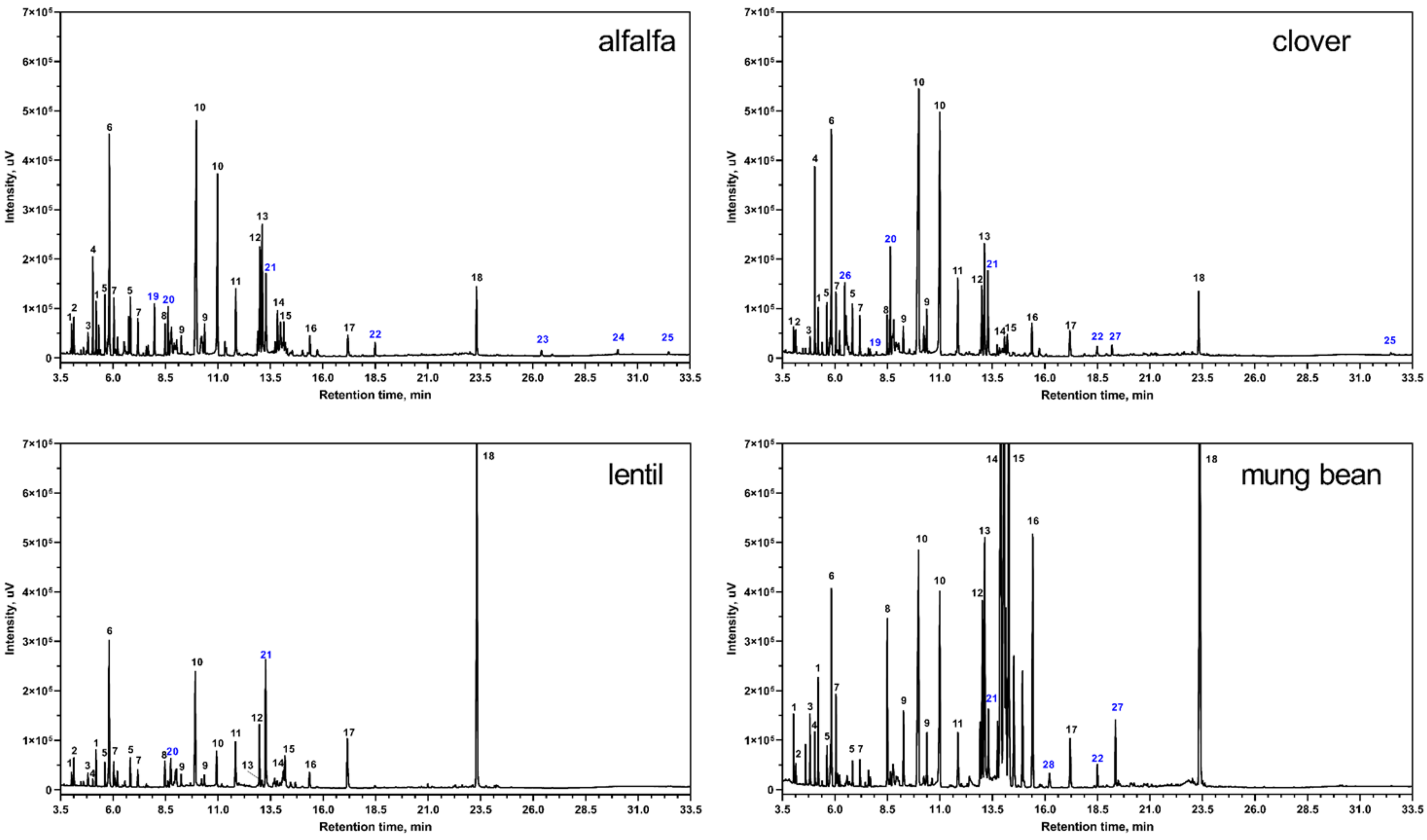

Abbreviations: 1 - valine; 2 - alanine; 3 - proline; 4 - malonic acid; 5 - serine; 6 - phosphoric acid; 7 - threonine; 8 - malic acid; 9 - phenylalanine; 10 - asparagine; 11 - ISDT (xylitol); 12 - citric acid; 13 - UNK1; 14 - fructose; 15 - glucose; 16 - UNK2; 17 - *myo*-inositol; 18 - sucrose; 19 - homoserine; 20 - UNK3; 21 - pinitol; 22 - tryptophan; 23 - galactinol; 24 - raffinose; 25 - ciceritol; 26 - propanoic acid; 27 - UNK4; 28 - *scyllo*-inositol.

Figure S2. The GC-chromatograms of TMS-derivatives of polar metabolites extracted from control sprouts of Fabaceae (alfalfa, clover, lentil and mung bean).

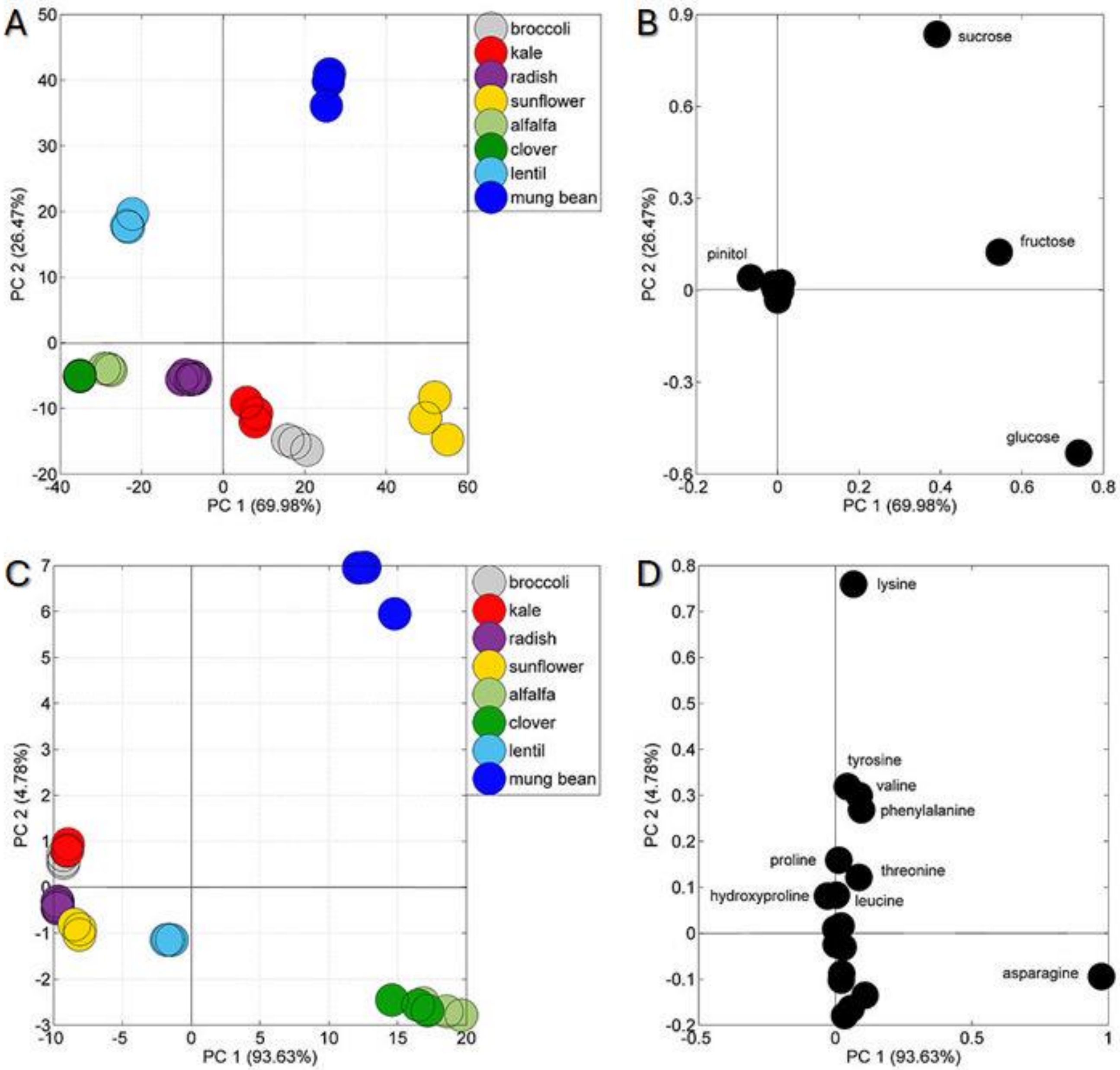

Figure S3. PCA score and loading plots of the soluble carbohydrate profiles (A, B) or proteinogenic amino acids profiles (C, D) of control sprouts of eight edible species.

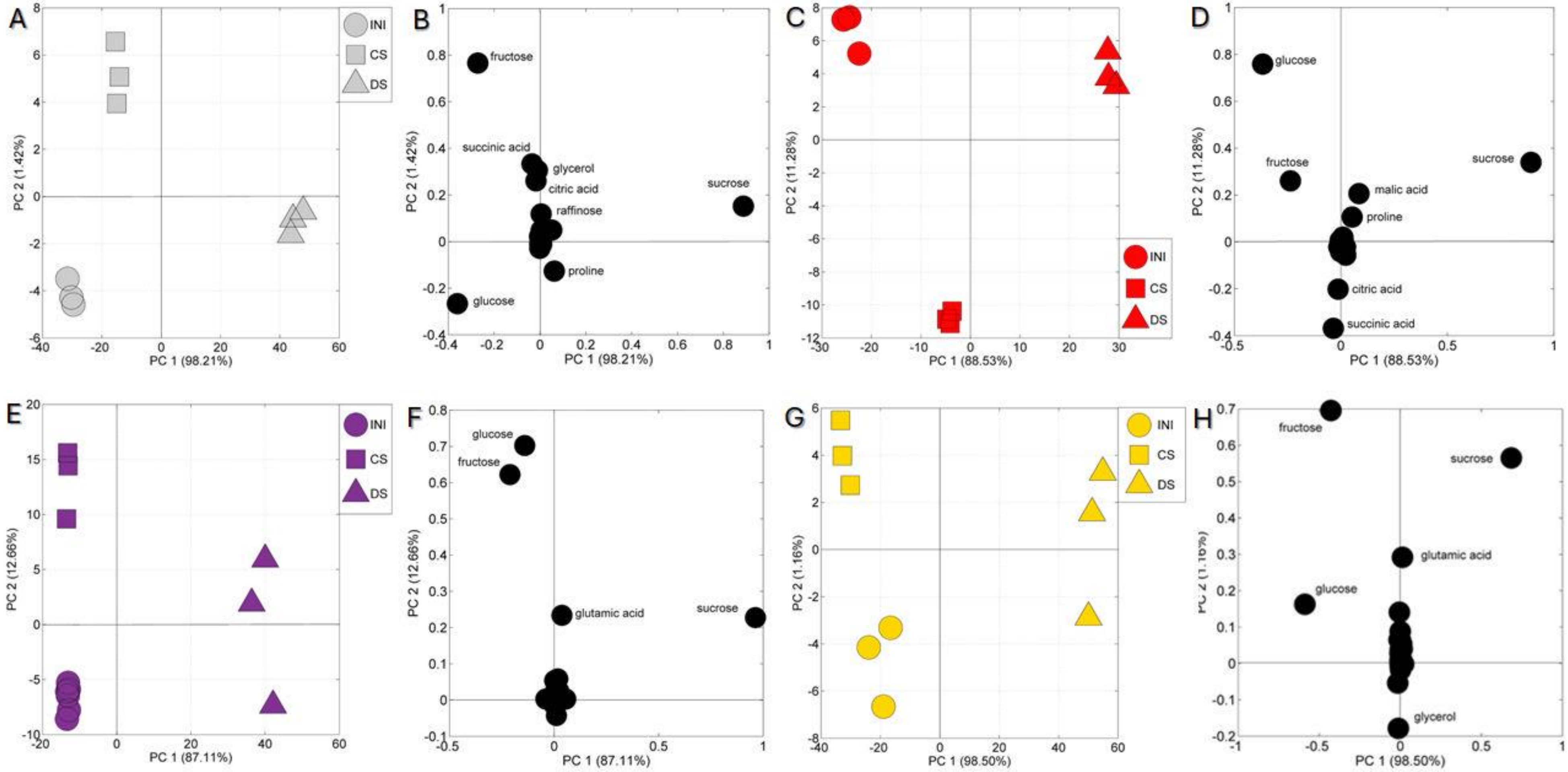

Figure S4. PCA score (A, C, E and G) and loading plots (B, D, F and H) of the polar metabolic profiles of broccoli (A, B), kale (C, D), radish (E, F) and sunflower (G, H) sprouts. Symbols: circles – control, squares – after 7 days of cold stress (CS), and triangles – after 7-10 days of drying (DS).

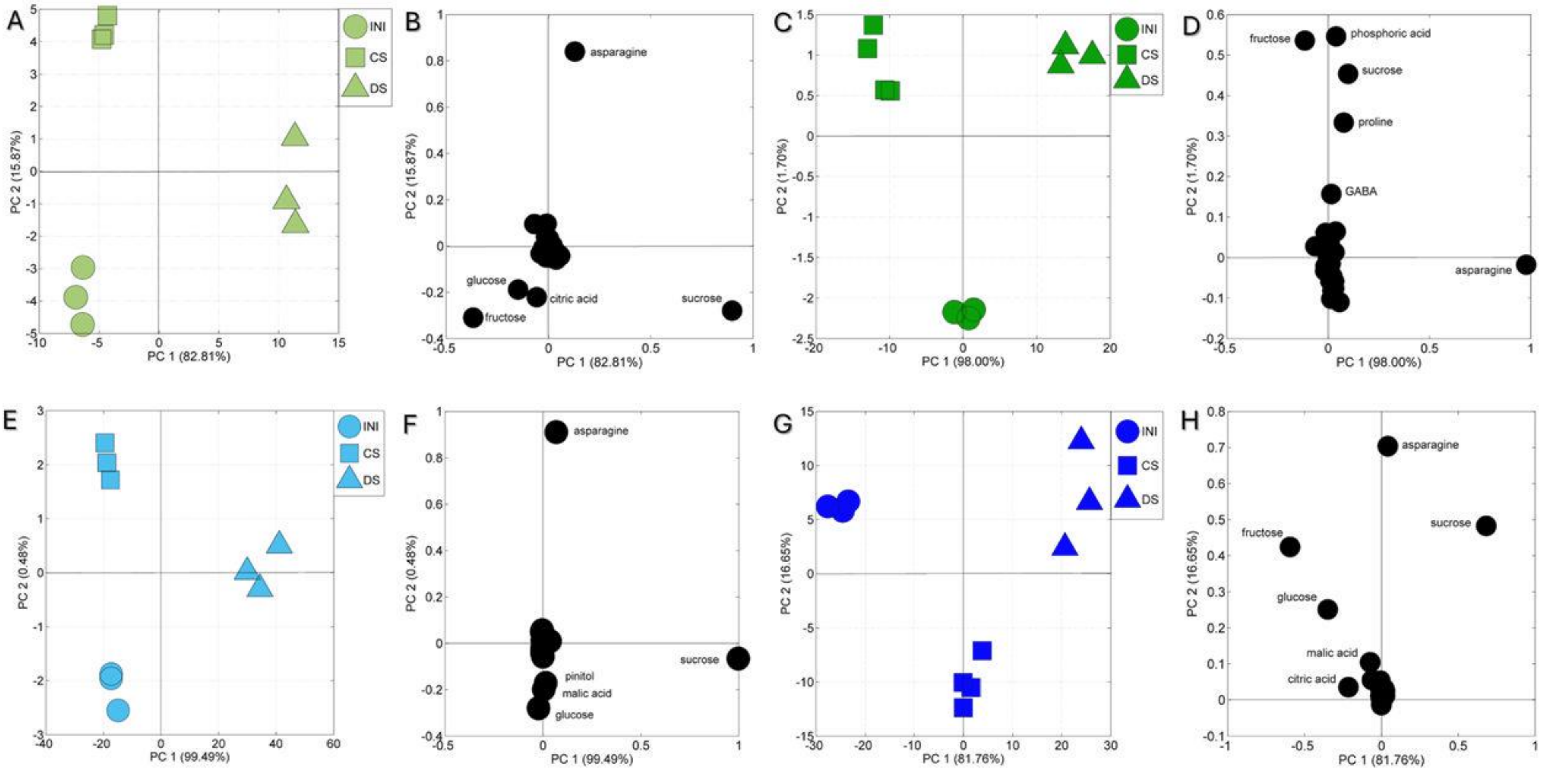

Figure S5. PCA score (A, C, E and G) and loading plots (B, D, F and H) of the polar metabolic profiles of alfalfa (A, B), clover (C, D), lentil (E, F) and mung bean (G, H) sprouts. Symbols: circles – control, squares – after 7 days of cold stress (CS), and triangles – after 7-10 days of drying (DS).
