# Supplementary material for: Cold Storage and Drying Alter Polar Metabolite Profiles in Commercial Sprouts of Eight Plant Species
Source: Molecules. 2026 Jul 12;31(14):2442. doi: 10.3390/molecules31142442 (PMC13415856; doi:10.3390/molecules31142442)
Supplement: Supplementary file 1 [file molecules-31-02442-s001.zip › Supplementary_Tables.pdf]

Table S1. The retention times (RTs) and relative retention times (RRTs) of identified polar metabolites.

Compound	Retention time (RT), min	Relative retention time (RTT)
**Internal standard - ribitol**	**11.853**	-
butyric acid	8.854	0.747
citric acid	13.000	1.097
chlorogenic acid	27.523	2.322
erythronic acid	9.549	0.806
fumaric acid	6.567	0.554
lactic acid	3.803	0.321
malic acid	8.486	0.716
oxalic acid	4.476	0.378
propanoic acid	6.477; 7.679	0.546; 0.648
pyroglutamic acid	8.573	0.723
succinic acid	6.217	0.525
synapic acid	18.880	1.593
glycerol	5.796	0.489
phosphoric acid	5.827	0.492
alanine	4.138	0.349
asparagine	9.937; 10.989	0.838; 0.927
aspartic acid	7.595; 8.913	0.641; 0.752
glutamic acid	8.978; 10.233	0.757; 0.863
glutamine	12.339	1.041
glycine	5.757; 6.219; 9.959	0.486; 0.525; 0.840
leucine	4.601	0.388
lysine	13.301; 14.294	1.122; 1.206
methionine	7.446	0.628
phenylalanine	9.259; 10.368	0.781; 0.875
proline	4.812; 6.116	0.406; 0.516
serine	5.621; 6.837	0.474; 0.577
threonine	6.048; 7.190	0.510; 0.607
tryptophan	18.492	1.560
tyrosine	13.732; 14.616	1.158; 1.233
valine	4.030; 5.200	0.340; 0.439
β-alanine	7.682	0.648
GABA	9.040	0.763
homoserine	6.757; 7.979	0.570; 0.673
hydroxyproline	8.964	0.756
ciceritol	32.468	2.739
D-*chiro*-inositol	15.027	1.268
D-pinitol	13.281	1.120
epi-inositol	16.027	1.352
fructose	13.861; 14.003	1.169; 1.181
galactinol	26.441	2.231
galactose	14.117; 14.369	1.191; 1.212
gluconic acid	15.766	1.330
glucose	14.252; 14.496	1.202; 1.223
glucuronic acid	15.012	1.266
maltose	24.170; 24.383	2.039; 2.057
myo-inositol	17.193	1.450
raffinose	30.052	2.535
scyllo-inositol	16.219	1.368
sorbitol	14.807	1.249
sucrose	23.347	1.970

Table S2. Concentration (mg/g DW ± SD) in sprouts of total polar metabolites (TPMs), including: total soluble carbohydrates (TSCs), total amino acids (TAAs), including: total essential amino acids (TEAAs) and total non-proteinogenic amino acids (TNPAAs), total organic acids (TOAs) and total other compounds (TOCs), in sprouts before (Initial, control) and after cold storage or after drying. The results marked with the same letters (compared separately in rows) indicate no significant differences (P < 0.05) after ANOVA and Tukey's test.

Family	Sprouts' species	Metabolites	Initial (control)	Cold storage	Drying
Brassicaceae	Broccoli (*Brassica oleracea* var. *italica)*	TPMs	119.1 ± 3.37[c]	140.2 ± 4.41[b]	153.5 ± 4.15[a]
		TSCs	92.17 ± 3.36[c]	104.1 ± 4.47[b]	116.9 ± 3.40[a]
		TAAs	10.52 ± 0.10[b]	10.71 ± 0.31[b]	17.67 ± 0.24[a]
		TEAAs	4.37 ± 0.04[a]	4.15 ± 0.15[a]	4.32 ± 0.04[a]
		TNPAAs	1.84 ± 0.17[c]	2.54 ± 0.22[b]	3.23 ± 0.34[a]
		TOAs	10.61 ± 0.20[c]	16.53 ± 0.47[a]	12.39 ± 0.63[b]
		TOCs	5.81 ± 0.20[c]	8.83 ± 0.28[a]	6.53 ± 0.23[b]
	Kale (*Brassica oleracea* var. *acephala)*	TPMs	103.5 ± 1.13[b]	98.20 ± 1.89[c]	125.9 ± 3.28[a]
		TSCs	74.76 ± 1.68[b]	58.04 ± 1.03[c]	88.08 ± 1.72[a]
		TAAs	12.55 ± 0.82[c]	15.09 ± 0.16[b]	18.31 ± 0.96[a]
		TEAAs	5.25 ± 0.22[b]	7.05 ± 0.40[a]	5.99 ± 0.13[b]
		TNPAAs	2.47 ± 0.29[b]	4.04 ± 0.37[a]	4.40 ± 0.76[a]
		TOAs	11.68 ± 1.07[c]	19.17 ± 0.86[a]	14.45 ± 0.84[c]
		TOCs	4.53 ± 0.24[c]	5.90 ± 0.06[a]	5.06 ± 0.01[b]
	Radish (*Raphanus sativus)*	TPMs	73.45 ± 2.90[b]	115.1 ± 7.68[a]	127.4 ± 9.62[a]
		TSCs	55.02 ± 2.27[b]	86.21 ± 5.24[a]	100.1 ± 9.34[a]
		TAAs	4.55 ± 0.20[b]	12.62 ± 1.56[a]	14.92 ± 0.69[a]
		TEAAs	2.10 ± 0.05[b]	4.16 ± 0.38[a]	4.22 ± 0.24[a]
		TNPAAs	0.05 ± 0.01[c]	0.19 ± 0.01[b]	0.54 ± 0.07[a]
		TOAs	7.41 ± 0.43[ab]	8.85 ± 0.66[a]	7.11 ± 0.86[b]
		TOCs	6.13 ± 0.23[b]	7.42 ± 0.43[a]	5.32 ± 0.17[c]
Asteraceae	Sunflower (*Helianthus annuus)*	TPMs	185.7 ± 7.08[b]	206.6 ± 4.75[a]	174.5 ± 7.41[b]
		TSCs	153.6 ± 5.14[b]	170.8 ± 3.71[a]	137.8 ± 5.46[b]
		TAAs	11.61 ± 0.90[b]	15.39 ± 0.88[a]	17.49 ± 1.31[a]
		TEAAs	2.89 ± 0.16[b]	3.07 ± 0.08[a]	3.48 ± 0.16[a]
		TNPAAs	1.14 ± 0.26[ab]	0.95 ± 0.01[b]	1.54 ± 0.21[a]
		TOAs	8.39 ± 1.01[a]	9.98 ± 0.36[a]	8.39 ± 0.53[a]
		TOCs	12.17 ± 0.62[a]	10.43 ± 0.20[b]	10.79 ± 0.46[b]
Fabaceae	Alfalfa (*Medicago sativa)*	TPMs	97.86 ± 4.04[b]	95.71 ± 8.80[b]	105.4 ± 1.04[a]
		TSCs	26.76 ± 1.41[b]	20.15 ± 0.64[c]	31.71 ± 1.20[a]
		TAAs	55.78 ± 2.76[b]	61.91 ± 0.65[a]	60.86 ± 1.96[a]
		TEAAs	10.34 ± 0.47[b]	11.85 ± 0.06[a]	11.49 ± 0.20[a]
		TNPAAs	4.48 ± 0.37[a]	3.97 ± 0.11[b]	4.77 ± 0.27[a]
		TOAs	7.52 ± 0.43[a]	6.41 ± 0.07[b]	4.90 ± 0.57[c]
		TOCs	7.78 ± 0.31[a]	7.24 ± 0.05[a]	7.95 ± 0.49[a]
	Clover (*Trifolium repens)*	TPMs	64.55 ± 2.38[b]	54.87 ± 0.97[c]	88.62 ± 3.46[a]
		TSCs	10.08 ± 0.42[b]	13.04 ± 0.75[a]	13.09 ± 0.56[a]
		TAAs	41.56 ± 1.77[b]	29.15 ± 1.06[c]	60.45 ± 2.84[a]
		TEAAs	7.33 ± 0.13[b]	6.30 ± 0.28[c]	8.91 ± 0.31[a]
		TNPAAs	0.54 ± 0.06[c]	1.07 ± 0.05[b]	1.40 ± 0.02[a]
		TOAs	7.45 ± 0.19[a]	5.87 ± 0.31[b]	7.31 ± 0.26[a]
		TOCs	5.36 ± 0.05[c]	6.81 ± 0.38[b]	7.77 ± 0.10[a]
	Lentil (*Lens culinaris)*	TPMs	84.29 ± 2.82[b]	83.10 ± 1.04[b]	142.10 ± 8.80[a]
		TSCs	49.24 ± 2.15[b]	44.86 ±1.13[b]	99.19 ± 6.50[a]
		TAAs	20.32 ± 0.29[c]	24.17 ± 0.27[b]	26.53 ± 1.40[a]

		TEAAs	4.76 ± 0.07[a]	4.83 ± 0.03[a]	4.48 ± 0.47[a]
		TNPAAs	0.64 ± 0.01[c]	0.52 ± 0.01[a]	0.32 ± 0.07[b]
		TOAs	5.36 ± 0.20[a]	4.68 ± 0.10[b]	4.93 ± 0.31[ab]
		TOCs	9.44 ± 0.23[b]	9.39 ± 0.01[b]	11.14 ± 0.60[a]
	Mung bean *(Vigna radiata)*	TPMs	219.83 ± 1.32[a]	167.31 ± 8.01[c]	197.74 ± 11.17[b]
		TSCs	129.06 ± 1.73[a]	102.90 ± 4.73[b]	118.11 ± 9.47[a]
		TAAs	53.87 ± 0.88[a]	37.81 ± 2.18[b]	55.52 ± 2.38[a]
		TEAAs	20.97 ± 0.21[a]	17.31 ±.1.38[b]	17.21 ± 0.76[b]
		TNPAAs	0.87 ± 0.16[c]	1.18 ± 0.07[b]	2.58 ± 0.05[a]
		TOAs	30.36 ± 0.10[a]	20.19 ± 0.90[b]	16.76 ± 0.62[c]
		TOCs	6.60 ± 0.32[ab]	6.41 ± 0.43[b]	7.34 ± 0.12[a]

Table S3. The concentration (mg/g DW ± SD) of polar metabolites in sprouts of *Brassicaceae* (broccoli, kale and radish) before (control) and after cold storage or after drying. The results marked with the same letters (compared in rows, separately for each species) indicate no significant differences ($P < 0.05$) after ANOVA and Tukey's test. bdl - below the detection limit

Metabolite	Broccoli			Kale			Radish		
	control	cold storage	drying	control	cold storage	drying	control	cold storage	drying
butyric acid	0.16 ± 0.03^{A}	0.16 ± 0.02^{A}	0.19 ± 0.02^{A}	bdl	bdl	bdl	bdl	bdl	bdl
citric acid	2.33 ± 0.19^{B}	4.61 ± 0.49^{A}	1.80 ± 0.27^{B}	2.71 ± 0.60^{B}	5.94 ± 0.09^{A}	2.52 ± 0.07^{B}	3.59 ± 0.30^{A}	3.68 ± 0.26^{A}	2.94 ± 0.59^{A}
erythronic acid	0.15 ± 0.01^{B}	0.21 ± 0.01^{A}	0.23 ± 0.03^{A}	0.15 ± 0.01^{C}	0.25 ± 0.01^{A}	0.18 ± 0.00^{B}	bdl	bdl	bdl
fumaric acid	bdl	bdl	bdl	0.08 ± 0.01^{B}	0.11 ± 0.01^{A}	0.10 ± 0.01^{AB}	0.03 ± 0.01^{C}	0.16 ± 0.01^{A}	0.12 ± 0.01^{B}
lactic acid	0.18 ± 0.00^{B}	0.20 ± 0.03^{AB}	0.33 ± 0.10^{A}	0.03 ± 0.00^{A}	0.38 ± 0.50^{A}	0.03 ± 0.00^{A}	0.02 ± 0.01^{C}	0.09 ± 0.01^{A}	0.06 ± 0.02^{B}
malic acid	4.99 ± 0.04^{C}	6.20 ± 0.13^{B}	8.98 ± 0.51^{A}	6.28 ± 0.34^{B}	4.38 ± 0.11^{C}	10.21 ± 0.91^{A}	3.07 ± 0.12^{C}	4.31 ± 0.36^{A}	3.59 ± 0.25^{B}
oxalic acid	0.12 ± 0.02^{A}	0.14 ± 0.00^{A}	0.11 ± 0.00^{A}	0.09 ± 0.01^{B}	0.24 ± 0.08^{A}	0.12 ± 0.01^{B}	0.04 ± 0.01^{B}	0.06 ± 0.00^{A}	0.07 ± 0.01^{A}
propanoic acid	0.64 ± 0.01^{A}	0.52 ± 0.00^{B}	0.51 ± 0.02^{B}	0.73 ± 0.05^{A}	0.56 ± 0.09^{B}	0.64 ± 0.02^{AB}	0.67 ± 0.03^{A}	0.54 ± 0.03^{B}	0.33 ± 0.02^{C}
pyroglutamic acid	0.05 ± 0.01^{B}	0.08 ± 0.00^{AB}	0.09 ± 0.02^{A}	bdl	bdl	bdl	bdl	bdl	bdl
succinic acid	1.99 ± 0.03^{B}	4.63 ± 0.13^{AB}	0.39 ± 0.00^{C}	1.32 ± 0.08^{B}	7.09 ± 0.08^{A}	0.40 ± 0.01^{C}	bdl	bdl	bdl
synapic acid	bdl	bdl	bdl	0.29 ± 0.00^{B}	0.47 ± 0.02^{A}	0.43 ± 0.02^{A}	bdl	bdl	bdl
glycerol	2.52 ± 0.07^{B}	5.22 ± 0.42^{A}	2.57 ± 0.14^{B}	1.96 ± 0.08^{A}	2.14 ± 0.22^{A}	1.50 ± 0.17^{B}	3.81 ± 0.18^{A}	3.90 ± 0.12^{A}	1.74 ± 0.17^{B}
phosphoric acid	3.29 ± 0.13^{B}	3.61 ± 0.39^{AB}	3.96 ± 0.12^{A}	2.57 ± 0.17^{B}	3.76 ± 0.26^{A}	3.57 ± 0.17^{A}	2.24 ± 0.15^{B}	3.52 ± 0.31^{A}	3.59 ± 0.26^{A}
alanine	0.22 ± 0.03^{B}	0.20 ± 0.00^{B}	0.27 ± 0.01^{A}	0.30 ± 0.02^{A}	0.23 ± 0.12^{A}	0.28 ± 0.00^{A}	0.18 ± 0.03^{C}	0.24 ± 0.03^{B}	0.31 ± 0.02^{A}
asparagine	0.28 ± 0.00^{C}	0.33 ± 0.00^{B}	0.77 ± 0.02^{A}	0.51 ± 0.03^{A}	0.54 ± 0.43^{A}	0.79 ± 0.01^{A}	0.03 ± 0.02^{C}	0.19 ± 0.02^{B}	0.25 ± 0.05^{A}
aspartic acid	0.17 ± 0.00^{B}	0.15 ± 0.00^{C}	0.21 ± 0.00^{A}	0.24 ± 0.01^{A}	0.16 ± 0.03^{B}	0.22 ± 0.00^{A}	0.35 ± 0.05^{A}	0.08 ± 0.00^{C}	0.19 ± 0.03^{B}
glutamic acid	0.76 ± 0.05^{B}	0.70 ± 0.04^{B}	0.98 ± 0.08^{A}	0.32 ± 0.12^{B}	0.13 ± 0.06^{B}	0.76 ± 0.17^{A}	0.10 ± 0.01^{C}	5.21 ± 0.96^{A}	3.77 ± 0.28^{B}
glutamine	bdl	bdl	0.40 ± 0.03	bdl	bdl	bdl	bdl	bdl	bdl
glycine	0.51 ± 0.04^{AB}	0.45 ± 0.04^{B}	0.59 ± 0.07^{A}	0.55 ± 0.02^{A}	0.86 ± 0.40^{A}	0.53 ± 0.00^{A}	0.12 ± 0.04^{B}	0.22 ± 0.01^{A}	0.23 ± 0.02^{A}
leucine	0.37 ± 0.02^{A}	0.18 ± 0.01^{C}	0.23 ± 0.00^{B}	0.46 ± 0.02^{A}	0.68 ± 0.34^{A}	0.33 ± 0.02^{A}	0.15 ± 0.01^{A}	0.14 ± 0.01^{AB}	0.12 ± 0.01^{B}
lysine	1.19 ± 0.09^{AB}	1.40 ± 0.11^{A}	1.11 ± 0.03^{B}	1.15 ± 0.05^{B}	1.84 ± 0.03^{A}	1.18 ± 0.01^{B}	0.47 ± 0.09^{A}	0.87 ± 0.31^{A}	0.85 ± 0.31^{A}
methionine	0.24 ± 0.01^{B}	0.19 ± 0.01^{C}	0.29 ± 0.00^{A}	0.23 ± 0.01^{A}	0.53 ± 0.48^{A}	0.25 ± 0.02^{A}	0.05 ± 0.01^{C}	0.10 ± 0.01^{B}	0.16 ± 0.02^{A}
phenylalanine	0.33 ± 0.03^{AB}	0.30 ± 0.02^{B}	0.40 ± 0.05^{A}	0.47 ± 0.02^{B}	0.62 ± 0.02^{A}	0.61 ± 0.05^{A}	0.00 ± 0.00^{C}	0.28 ± 0.02^{B}	0.31 ± 0.00^{A}
proline	1.30 ± 0.05^{B}	1.05 ± 0.01^{B}	5.62 ± 0.21^{A}	1.43 ± 0.07^{B}	0.61 ± 0.56^{B}	3.96 ± 0.05^{A}	0.86 ± 0.04^{B}	0.88 ± 0.07^{B}	3.79 ± 0.15^{A}
serine	0.72 ± 0.08^{B}	0.81 ± 0.01^{B}	0.95 ± 0.03^{A}	1.02 ± 0.05^{A}	1.05 ± 0.03^{A}	0.90 ± 0.42^{A}	0.65 ± 0.03^{C}	1.00 ± 0.12^{B}	1.17 ± 0.04^{A}
threonine	1.18 ± 0.10^{A}	1.15 ± 0.04^{A}	1.18 ± 0.05^{A}	1.40 ± 0.06^{A}	1.57 ± 0.29^{A}	1.63 ± 0.03^{A}	0.64 ± 0.03^{B}	1.55 ± 0.19^{A}	1.46 ± 0.13^{A}
tryptophan	bdl	bdl	bdl	0.20 ± 0.00^{B}	0.46 ± 0.02^{A}	0.46 ± 0.09^{A}	0.22 ± 0.02^{A}	0.21 ± 0.02^{A}	0.19 ± 0.02^{A}
tyrosine	0.32 ± 0.04^{A}	0.33 ± 0.04^{A}	0.33 ± 0.00^{A}	0.45 ± 0.03^{A}	0.43 ± 0.01^{A}	0.48 ± 0.04^{A}	0.00 ± 0.00^{B}	0.46 ± 0.11^{A}	0.44 ± 0.06^{A}
valine	1.06 ± 0.03^{A}	0.93 ± 0.01^{B}	1.11 ± 0.04^{A}	1.34 ± 0.06^{A}	1.35 ± 0.23^{A}	1.53 ± 0.02^{A}	0.59 ± 0.03^{C}	1.00 ± 0.07^{B}	1.13 ± 0.04^{A}
β-alanine	0.04 ± 0.01^{A}	0.06 ± 0.00^{A}	0.05 ± 0.01^{A}	bdl	bdl	bdl	bdl	bdl	bdl
GABA	0.29 ± 0.02^{B}	0.30 ± 0.02^{B}	0.68 ± 0.03^{A}	0.21 ± 0.00^{C}	0.31 ± 0.00^{B}	0.78 ± 0.05^{A}	0.05 ± 0.01^{C}	0.19 ± 0.01^{B}	0.54 ± 0.07^{A}

hydroxyproline	1.51 ± 0.17^{B}	2.19 ± 0.22^{A}	2.51 ± 0.32^{A}	2.26 ± 0.29^{B}	3.74 ± 0.37^{A}	3.62 ± 0.74^{A}	bdl	bdl	bdl
fructose	30.85 ± 1.46^{A}	33.48 ± 2.17^{A}	12.51 ± 0.39^{B}	22.35 ± 0.97^{A}	13.12 ± 0.15^{B}	9.27 ± 0.87^{C}	17.91 ± 0.88^{B}	30.42 ± 2.05^{A}	11.02 ± 4.16^{C}
galactinol	0.04 ± 0.01^{B}	0.12 ± 0.01^{A}	0.12 ± 0.01^{A}	0.07 ± 0.01^{B}	0.07 ± 0.00^{B}	0.13 ± 0.01^{A}	0.02 ± 0.00^{B}	0.21 ± 0.02^{A}	0.18 ± 0.02^{A}
galactose	0.88 ± 0.51^{A}	1.67 ± 0.72^{A}	1.90 ± 0.39^{A}	bdl	bdl	bdl	4.54 ± 0.22^{A}	3.90 ± 0.13^{A}	4.83 ± 1.44^{A}
gluconic acid	0.09 ± 0.02^{C}	0.53 ± 0.04^{B}	0.87 ± 0.02^{A}	0.24 ± 0.02^{A}	0.06 ± 0.01^{B}	0.05 ± 0.01^{B}	bdl	bdl	bdl
glucose	44.76 ± 2.09^{A}	36.06 ± 2.16^{B}	16.41 ± 0.44^{C}	35.76 ± 1.52^{A}	15.2 ± 0.31^{B}	14.58 ± 1.11^{B}	19.74 ± 0.91^{B}	33.45 ± 2.06^{A}	17.10 ± 5.92^{B}
glucuronic acid	0.31 ± 0.02^{A}	0.03 ± 0.02^{C}	0.17 ± 0.05^{B}	bdl	bdl	bdl	bdl	bdl	bdl
myo-inositol	1.62 ± 0.02^{B}	2.15 ± 0.04^{A}	2.13 ± 0.07^{A}	1.31 ± 0.08^{B}	1.56 ± 0.05^{A}	1.60 ± 0.02^{A}	0.87 ± 0.07^{B}	1.33 ± 0.10^{A}	1.41 ± 0.09^{A}
raffinose	0.06 ± 0.03^{C}	1.29 ± 0.01^{A}	0.82 ± 0.06^{B}	0.11 ± 0.02^{C}	0.94 ± 0.07^{B}	1.32 ± 0.20^{A}	0.08 ± 0.05^{C}	0.67 ± 0.02^{B}	1.43 ± 0.12^{A}
sorbitol	bdl	bdl	0.49 ± 0.01	bdl	bdl	bdl	bdl	bdl	bdl
sucrose	13.57 ± 0.17^{C}	28.59 ± 0.99^{B}	81.25 ± 2.62^{A}	14.91 ± 0.99^{C}	26.85 ± 0.70^{B}	60.95 ± 0.53^{A}	11.84 ± 0.44^{C}	16.22 ± 0.92^{B}	64.11 ± 2.37^{A}

Table S4. The concentration (mg/g DW ± SD) of polar metabolites before (control) and after cold storage or after drying of sunflower sprouts. The results marked with the same letters (compared in rows, separately for each species) indicate no significant differences (P < 0.05) after ANOVA and Tukey's test. bdl - below the detection limit

Metabolite	Sunflower		
	control	cold storage	drying
chlorogenic acid	2.05 ± 0.50^{B}	3.68 ± 0.15^{A}	2.58 ± 0.21^{B}
citric acid	1.63 ± 1.10^{A}	1.74 ± 0.04^{A}	2.56 ± 0.46^{A}
fumaric acid	0.81 ± 0.12^{A}	0.84 ± 0.08^{A}	0.67 ± 0.09^{A}
lactic acid	0.04 ± 0.02^{B}	0.38 ± 0.01^{A}	0.07 ± 0.01^{B}
malic acid	3.73 ± 0.23^{A}	3.16 ± 0.10^{A}	2.12 ± 0.32^{B}
oxalic acid	0.05 ± 0.01^{B}	0.04 ± 0.01^{B}	0.10 ± 0.01^{A}
propanoic acid	0.07 ± 0.01^{C}	0.14 ± 0.00^{B}	0.29 ± 0.03^{A}
glycerol	6.94 ± 0.25^{A}	4.95 ± 0.13^{B}	4.73 ± 0.18^{B}
phosphoric acid	5.22 ± 0.37^{B}	5.48 ± 0.09^{AB}	6.06 ± 0.29^{A}
alanine	0.35 ± 0.04^{C}	0.76 ± 0.06^{A}	0.57 ± 0.03^{B}
asparagine	1.41 ± 0.20^{B}	1.88 ± 0.06^{A}	1.90 ± 0.08^{A}
aspartic acid	0.30 ± 0.02^{A}	0.32 ± 0.01^{A}	0.17 ± 0.00^{B}
glutamic acid	2.25 ± 0.57^{B}	4.65 ± 0.71^{A}	4.76 ± 1.29^{A}
glycine	0.54 ± 0.03^{B}	0.66 ± 0.02^{A}	0.47 ± 0.02^{C}
leucine	0.44 ± 0.01^{A}	0.35 ± 0.04^{B}	0.39 ± 0.02^{AB}
methionine	0.20 ± 0.01^{A}	0.13 ± 0.00^{A}	0.16 ± 0.05^{A}
phenylalanine	0.18 ± 0.01^{B}	0.23 ± 0.01^{A}	0.23 ± 0.01^{A}
proline	1.65 ± 0.03^{B}	1.36 ± 0.04^{C}	3.09 ± 0.08^{A}
serine	0.84 ± 0.09^{C}	1.52 ± 0.03^{A}	1.28 ± 0.03^{B}
threonine	0.72 ± 0.04^{B}	0.94 ± 0.04^{A}	0.95 ± 0.05^{A}
tryptophan	0.46 ± 0.13^{A}	0.46 ± 0.03^{A}	0.48 ± 0.09^{A}
tyrosine	0.24 ± 0.00^{A}	0.21 ± 0.00^{B}	0.21 ± 0.01^{B}
valine	0.89 ± 0.04^{B}	0.96 ± 0.01^{B}	1.27 ± 0.07^{A}
GABA	0.38 ± 0.02^{B}	0.44 ± 0.02^{B}	0.82 ± 0.08^{A}
hydroxyproline	0.76 ± 0.24^{A}	0.51 ± 0.03^{A}	0.72 ± 0.15^{A}
fructose	43.08 ± 1.35^{B}	55.00 ± 0.93^{A}	16.34 ± 0.84^{C}
galactose	3.32 ± 2.14^{A}	4.90 ± 0.59^{A}	3.54 ± 0.48^{A}
glucose	71.46 ± 3.21^{B}	78.96 ± 2.68^{A}	29.28 ± 1.61^{C}
myo-inositol	2.09 ± 0.12^{B}	2.42 ± 0.07^{A}	2.57 ± 0.13^{A}
raffinose	0.10 ± 0.03^{B}	0.13 ± 0.00^{B}	0.69 ± 0.21^{A}
sucrose	33.51 ± 2.62^{B}	29.38 ± 0.48^{B}	85.42 ± 4.10^{A}

Table S5. The concentration (mg/g DW ± SD) of polar metabolites in sprouts of legumes (alfalfa, clover, lentil and mung bean) before (control) and after cold storage or after drying. The results marked with the same letters (compared in rows, separately for each species) indicate no significant differences ($P < 0.05$) after ANOVA and Tukey's test. bdl - below the detection limit

Metabolite	Alfalfa			Clover		
	control	cold storage	drying	control	cold storage	drying
citric acid	4.74 ± 0.44 A	2.74 ± 0.12 B	2.94 ± 0.41 B	2.25 ± 0.06 A	1.75 ± 0.02 C	2.14 ± 0.02 B
fumaric acid	bdl	bdl	bdl	1.17 ± 0.05 A	0.65 ± 0.16 B	1.19 ± 0.14 A
lactic acid	0.11 ± 0.00 B	0.43 ± 0.03 A	0.03 ± 0.03 C	0.06 ± 0.00 B	0.07 ± 0.01 A	0.08 ± 0.01 A
malic acid	2.42 ± 0.07 B	3.11 ± 0.03 A	1.53 ± 0.08 C	2.02 ± 0.04 A	2.07 ± 0.12 A	1.68 ± 0.10 B
oxalic acid	0.18 ± 0.02 A	0.14 ± 0.00 A	0.40 ± 0.23 A	0.14 ± 0.00 B	0.13 ± 0.01 B	0.23 ± 0.02 A
propanoic acid	bdl	bdl	bdl	1.81 ± 0.08 A	1.20 ± 0.06 B	1.99 ± 0.10 A
erythronic acid	0.07 ± 0.02 B	0.09 ± 0.00 B	0.12 ± 0.00 A	0.10 ± 0.01 A	0.08 ± 0.01 B	0.10 ± 0.01 AB
glycerol	0.66 ± 0.07 A	0.53 ± 0.02 AB	0.38 ± 0.18 B	0.42 ± 0.02 B	0.69 ± 0.05 A	0.51 ± 0.11 B
phosphoric acid	7.12 ± 0.26 AB	6.71 ± 0.06 B	7.57 ± 0.33 A	4.94 ± 0.03 C	6.12 ± 0.33 B	7.26 ± 0.20 A
alanine	1.43 ± 0.08 A	1.35 ± 0.02 A	1.40 ± 0.05 A	0.67 ± 0.02 B	0.88 ± 0.05 A	0.56 ± 0.01 C
asparagine	27.25 ± 1.34 B	34.23 ± 0.40 A	32.27 ± 1.61 A	25.68 ± 1.35 B	14.14 ± 1.51 C	39.89 ± 2.41 A
aspartic acid	0.95 ± 0.08 A	0.83 ± 0.01 A	0.46 ± 0.09 B	0.60 ± 0.04 A	0.57 ± 0.09 A	0.57 ± 0.01 A
glutamic acid	2.28 ± 0.11 A	1.90 ± 0.02 B	1.47 ± 0.03 C	1.65 ± 0.09 A	1.06 ± 0.18 B	1.86 ± 0.09 A
glycine	1.36 ± 0.08 A	1.27 ± 0.01 A	1.13 ± 0.17 A	1.07 ± 0.05 A	0.65 ± 0.08 B	1.10 ± 0.06 A
leucine	0.23 ± 0.02 A	0.22 ± 0.01 A	0.18 ± 0.04 A	0.13 ± 0.01 B	0.24 ± 0.03 A	0.13 ± 0.01 B
lysine	0.71 ± 0.03 B	1.49 ± 0.08 A	0.87 ± 0.19 B	0.20 ± 0.01 B	bdl	0.34 ± 0.04 A
methionine	0.22 ± 0.08 A	0.19 ± 0.01 A	0.14 ± 0.03 A	0.09 ± 0.01 B	0.05 ± 0.01 C	0.13 ± 0.03 A
phenylalanine	2.03 ± 0.11 B	2.29 ± 0.02 A	2.16 ± 0.06 AB	2.03 ± 0.03 B	1.75 ± 0.15 C	2.75 ± 0.09 A
proline	1.40 ± 0.06 B	1.15 ± 0.00 B	2.14 ± 0.21 A	0.77 ± 0.01 B	0.88 ± 0.10 B	2.97 ± 0.07 A
serine	5.00 ± 0.24 A	4.77 ± 0.13 A	5.14 ± 0.17 A	2.92 ± 0.04 B	3.23 ± 0.18 A	2.90 ± 0.04 B
threonine	3.37 ± 0.17 A	3.40 ± 0.09 A	3.62 ± 0.00 A	2.43 ± 0.12 B	2.07 ± 0.15 C	2.91 ± 0.08 A
tryptophan	1.36 ± 0.09 A	1.18 ± 0.00 B	1.16 ± 0.03 B	0.73 ± 0.09 A	0.44 ± 0.08 B	0.69 ± 0.15 A
tyrosine	0.71 ± 0.02 A	0.61 ± 0.10 B	0.57 ± 0.05 B	0.33 ± 0.01 C	0.37 ± 0.03 C	0.29 ± 0.03 C
valine	3.02 ± 0.16 B	3.08 ± 0.04 AB	3.36 ± 0.14 A	1.73 ± 0.04 B	1.75 ± 0.09 B	1.95 ± 0.07 A
β-alanine	0.45 ± 0.02 A	0.46 ± 0.00 A	0.47 ± 0.02 A	0.17 ± 0.01 B	0.17 ± 0.02 B	0.21 ± 0.01 A
GABA	0.44 ± 0.04 B	0.38 ± 0.00 B	0.88 ± 0.03 A	0.22 ± 0.03 C	0.53 ± 0.02 B	0.95 ± 0.02 A
homoserine	3.59 ± 0.32 A	3.12 ± 0.10 A	3.42 ± 0.22 A	0.16 ± 0.03 B	0.37 ± 0.05 A	0.24 ± 0.02 B
ciceritol	0.23 ± 0.05 A	0.32 ± 0.04 A	0.29 ± 0.03 A	0.20 ± 0.05 B	0.37 ± 0.05 A	0.34 ± 0.07 A
D-*chiro*-inositol	0.46 ± 0.07 B	0.62 ± 0.06 A	0.60 ± 0.02 A	bdl	bdl	bdl
D-pinitol	4.67 ± 0.07 A	4.82 ± 0.25 A	4.78 ± 0.08 A	3.56 ± 0.18 A	2.49 ± 0.21 B	3.98 ± 0.16 A
epi-inositol	bdl	bdl	bdl	0.11 ± 0.01 A	0.05 ± 0.00 B	0.11 ± 0.01 A

fructose	9.10 ± 0.91 A	5.59 ± 0.30 AB	1.43 ± 0.46 B	0.62 ± 0.07 B	3.68 ± 0.30 A	0.61 ± 0.27 B
galactinol	0.51 ± 0.03 A	0.05 ± 0.01 C	0.13 ± 0.01 B	0.04 ± 0.01 B	0.01 ± 0.00 C	0.08 ± 0.00 A
galactose	1.81 ± 0.01 A	1.64 ± 0.05 B	1.81 ± 0.11 AB	bdl	bdl	bdl
gluconic acid	0.65 ± 0.11 B	0.68 ± 0.02 B	0.88 ± 0.03 A	0.69 ± 0.08 B	0.28 ± 0.03 C	0.83 ± 0.05 A
glucose	3.56 ± 0.45 A	1.58 ± 0.10 AB	0.24 ± 0.05 B	1.19 ± 0.01 B	2.10 ± 0.31 A	0.35 ± 0.16 C
maltose	0.06 ± 0.01 B	0.05 ± 0.00 B	0.16 ± 0.02 A	bdl	bdl	0.09 ± 0.02 A
myo-inositol	1.19 ± 0.04 A	1.00 ± 0.04 B	0.98 ± 0.03 B	0.99 ± 0.02 A	1.07 ± 0.09 A	0.71 ± 0.02 B
raffinose	0.37 ± 0.02 B	0.12 ± 0.03 C	1.23 ± 0.06 A	bdl	0.02 ± 0.00 B	0.33 ± 0.01 A
scyllo-inositol	bdl	bdl	bdl	bdl	bdl	bdl
sucrose	4.15 ± 0.11 B	3.58 ± 0.08 B	19.07 ± 0.58 A	2.68 ± 0.12 B	2.87 ± 0.21 B	5.55 ± 0.15 A

Table S5. *Continued*

**Metabolite**	**Lentil**			**Mung bean**		
	**control**	**cold storage**	**drying**	**control**	**cold storage**	**drying**
citric acid	2.92 ± 0.12 AB	3.18 ± 0.09 A	2.80 ± 0.17 B	17.88 ± 0.41 A	11.65 ± 0.53 B	7.38 ± 0.35 C
erythronic acid	0.04 ± 0.01 B	0.03 ± 0.00 B	0.07 ± 0.01 A	0.06 ± 0.01 B	0.07 ± 0.01 B	0.11 ± 0.01 A
lactic acid	0.04 ± 0.00 A	0.02 ± 0.01 A	0.04 ± 0.01 A	0.05 ± 0.00 B	0.07 ± 0.01 B	0.26 ± 0.02 A
malic acid	2.21 ± 0.07 A	1.35 ± 0.02 C	1.92 ± 0.11 B	11.46 ± 0.43 A	7.69 ± 0.43 B	7.95 ± 0.28 B
oxalic acid	0.15 ± 0.02 A	0.13 ± 0.01 A	0.16 ± 0.02 A	0.07 ± 0.03 B	0.10 ± 0.01 AB	0.14 ± 0.02 A
propanoic acid	bdl	bdl	bdl	0.08 ± 0.01 B	0.07 ± 0.01 B	0.11 ± 0.02 A
succinic acid	bdl	bdl	bdl	0.76 ± 0.05 B	0.60 ± 0.04 C	0.92 ± 0.01 A
glycerol	4.72 ± 0.11 B	4.70 ± 0.01 B	5.57 ± 0.30 A	1.15 ± 0.14 A	0.75 ± 0.09 B	0.83 ± 0.12 B
phosphoric acid	4.72 ± 0.11 B	4.70 ± 0.01 B	5.57 ± 0.30 A	5.45 ± 0.18 B	5.66 ± 0.41 B	6.51 ± 0.24 A
alanine	0.99 ± 0.05 A	0.75 ± 0.01 B	0.85 ± 0.05 B	0.73 ± 0.05 A	0.71 ± 0.09 A	0.84 ± 0.04 A
asparagine	7.80 ± 0.22 C	11.45 ± 0.30 B	13.28 ± 0.88 A	21.63 ± 1.48 B	10.63 ± 0.28 C	24.63 ± 1.42 A
aspartic acid	0.36 ± 0.01 A	0.39 ± 0.01 A	0.41 ± 0.07 A	0.86 ± 0.00 B	0.70 ± 0.04 C	1.00 ± 0.08 A
glutamic acid	1.34 ± 0.12 AB	1.59 ± 0.14 A	1.09 ± 0.07 B	0.35 ± 0.05 B	0.49 ± 0.10 AB	0.55 ± 0.08 A
glycine	0.77 ± 0.03 B	0.88 ± 0.02 A	0.92 ± 0.05 A	0.25 ± 0.03 A	0.16 ± 0.03 B	0.30 ± 0.05 A
leucine	0.15 ± 0.00 A	0.16 ± 0.01 A	0.09 ± 0.02 B	0.93 ± 0.13 AB	1.12 ± 0.11 A	0.87 ± 0.05 B
lysine	bdl	bdl	bdl	7.17 ± 0.49 A	4.70 ± 0.86 B	4.15 ± 0.28 B
methionine	0.05 ± 0.00 A	0.05 ± 0.02 A	0.04 ± 0.01 A	0.20 ± 0.05 A	0.14 ± 0.01 A	0.18 ± 0.04 A
phenylalanine	0.95 ± 0.01 A	0.84 ± 0.03 A	0.66 ± 0.35 A	4.20 ± 0.14 A	3.61 ± 0.28 A	4.00 ± 0.32 A
proline	1.01 ± 0.04 B	0.99 ± 0.07 B	2.93 ± 0.13 A	2.56 ± 0.34 B	2.55 ± 0.19 B	3.50 ± 0.17 A
serine	2.24 ± 0.12 A	2.42 ± 0.04 A	2.33 ± 0.14 A	2.28 ± 0.04 A	1.93 ± 0.15 B	2.11 ± 0.04 AB
threonine	1.51 ± 0.05 A	1.58 ± 0.04 B	1.37 ± 0.02 A	3.57 ± 0.15 A	2.97 ± 0.20 B	3.33 ± 0.11 AB
tryptophan	0.30 ± 0.02 B	0.44 ± 0.01 A	0.45 ± 0.01 A	0.42 ± 0.18 A	0.76 ± 0.03 B	1.50 ± 0.10 AB

tyrosine	0.39 ± 0.01 A	0.35 ± 0.01 B	0.23 ± 0.05 A	3.35 ± 0.53 A	2.16 ± 0.44 A	2.80 ± 0.14 A
valine	1.80 ± 0.02 A	1.77 ± 0.01 A	1.88 ± 0.10 A	4.90 ± 0.13 A	4.77 ± 0.34 A	4.68 ± 0.14 A
β-alanine	0.06 ± 0.00 B	0.07 ± 0.00 A	0.08 ± 0.00 A	0.32 ± 0.01 A	0.20 ± 0.03 C	0.26 ± 0.01 B
GABA	0.55 ± 0.02 A	0.42 ± 0.01 B	0.22 ± 0.07 C	0.13 ± 0.01 C	0.22 ± 0.02 B	0.82 ± 0.05 A
homoserine	0.03 ± 0.01 A	0.03 ± 0.01 A	0.02 ± 0.00 B	bdl	bdl	bdl
hydroxyproline	bdl	bdl	bdl	0.42 ± 0.18 C	0.76 ± 0.03 B	1.50 ± 0.10 A
ciceritol	0.08 ± 0.00 A	0.08 ± 0.00 A	0.09 ± 0.02 A	0.06 ± 0.01 B	0.09 ± 0.01 A	0.11 ± 0.01 A
D-*chiro*-inositol	0.03 ± 0.00 B	0.04 ± 0.00 AB	0.05 ± 0.01 A	bdl	bdl	bdl
D-pinitol	7.46 ± 0.27 B	6.70 ± 0.11 B	7.74 ± 0.42 A	bdl	bdl	bdl
epi-inositol	2.83 ± 0.11 A	2.82 ± 0.06 A	2.86 ± 0.20 A	bdl	bdl	bdl
fructose	0.76 ± 0.05 A	0.58 ± 0.03 B	0.42 ± 0.04 C	44.57 ± 0.26 A	22.31 ± 0.73 B	15.72 ± 3.12 B
galactinol	bdl	bdl	bdl	0.08 ± 0.01 B	0.31 ± 0.02 A	0.02 ± 0.00 C
galactose	0.93 ± 0.03 C	1.13 ± 0.01 A	1.01 ± 0.00 B	20.08 ± 0.69 B	6.89 ± 0.72 B	3.23 ± 1.26 A
gluconic acid	0.14 ± 0.01 B	0.12 ± 0.03 B	0.19 ± 0.02 A	0.23 ± 0.02 B	0.15 ± 0.02 B	1.04 ± 0.08 A
glucose	2.67 ± 0.28 A	1.53 ± 0.08 B	0.84 ± 0.04 C	bdl	bdl	0.89 ± 0.12 B
maltose	0.16 ± 0.03 B	0.07 ± 0.01 C	0.28 ± 0.03 A	bdl	bdl	bdl
myo-inositol	2.83 ± 0.11 A	2.82 ± 0.06 A	2.86 ± 0.20 A	2.31 ± 0.06 A	1.15 ± 0.07 C	1.97 ± 0.06 B
raffinose	0.03 ± 0.01 B	0.03 ± 0.00 B	0.14 ± 0.02 A	0.09 ± 0.01 B	0.02 ± 0.01 C	0.21 ± 0.00 A
scyllo-inositol	bdl	bdl	bdl	0.70 ± 0.05 A	0.27 ± 0.01 B	0.66 ± 0.00 A
sucrose	31.32 ± 1.42 B	28.96 ± 0.99 B	82.71 ± 5.59 A	60.88 ± 2.66 C	71.62 ± 3.54 B	94.16 ± 5.70 A
